# Interactions between the DNA Damage Response and the Telomere Complex in Carcinogenesis: A Hypothesis

**DOI:** 10.3390/cimb45090478

**Published:** 2023-09-19

**Authors:** Antonio Torres-Montaner

**Affiliations:** 1 Department of Pathology, Queen’s Hospital, Rom Valley Way, Romford, London RM7 OAG, UK; atorresmontaner@gmail.com; 2 Departamento de Bioquímica y Biologia Molecular, Universidad de Cadiz, Puerto Real, 11510 Cadiz, Spain

**Keywords:** telomeres, telomerase, DNA damage response (DDR), replication stress, cancer stem cell

## Abstract

Contrary to what was once thought, direct cancer originating from normal stem cells seems to be extremely rare. This is consistent with a preneoplastic period of telomere length reduction/damage in committed cells that becomes stabilized in transformation. Multiple observations suggest that telomere damage is an obligatory step preceding its stabilization. During tissue turnover, the telomeres of cells undergoing differentiation can be damaged as a consequence of defective DNA repair caused by endogenous or exogenous agents. This may result in the emergence of new mechanism of telomere maintenance which is the final outcome of DNA damage and the initial signal that triggers malignant transformation. Instead, transformation of stem cells is directly induced by primary derangement of telomere maintenance mechanisms. The newly modified telomere complex may promote survival of cancer stem cells, independently of telomere maintenance. An inherent resistance of stem cells to transformation may be linked to specific, robust mechanisms that help maintain telomere integrity.

## 1. Introduction

In a previous paper (1), I discussed the possible roles played by the alteration of the telomere complex in the initiation of malignant transformation. An extensive survey of the literature showed that the most common cancers display markers of differentiation and some reduction of telomere length [1]. This suggests that common adult cancers arise from cells that, even if they are not terminally differentiated, are in the process of differentiation and concomitant telomere erosion. Therefore, the carcinoma in situ stage, a typical feature of common adult cancers, seems to be reached at a critical threshold of telomere erosion [2]. However, tumors exhibiting an undifferentiated/immature phenotype and sudden onset that apparently precludes sustained gradual telomere erosion, such as early childhood cancers, acute leukemia and some sarcomas, do not appear to develop through a carcinoma in situ stage, but even in these tumors a substantial degree of telomere erosion is usually observed [3,4], suggesting a former process of telomere length reduction before malignant transformation stabilizes telomere length. Consistent with this concept, it has been estimated that premature telomere loss precedes leukemia onset for years [3] and that the rate of telomere length reduction is especially high in the first years of life probably due to a high proliferation rate [5]. The period of telomere erosion prior to transformation suggests that cancer transformation does not occur during the stem cell stage.

The availability of markers for every stage of differentiation of hematopoietic development allows for the assignment of a precise stage of origin to most hematological cancers, and common adult cancers also exhibit differentiation markers expressed by normal counterpart cells at stages of differentiation downstream of the stem cell stage [6]. The phenotype of the cancer cell of mature as well as immature tumors can usually be ascribed to a definite developmental stage. Instead, cancer cells with an unmistakable stem cell phenotype are extremely rare. This appears to be true for common adult cancers but also appears to be the case for most leukemia, lymphomas, and tumors of myeloid origin.

The aforementioned survey [1] highlighted a difference in cancer pathways between tumors of putative stem cell origin and more differentiated/mature tumors: a wide spectrum of diverse cancer-driving mutations that enhance proliferation/decrease apoptosis is usually identified in common adult/mature tumors, whereas pathways that can directly affect telomere maintenance drove transformation of true stem cell/early progenitor tumors. One instance taken from the human clinic in support of this contention is the Beckwith–Wiedemann syndrome, a genetic disease of the telomerase reverse transcriptase (TERT)-TGFβ-Smad pathway which is associated with an 800-fold risk of developing childhood cancer in many different organs [7]. Another is represented by the tumor model reported by Passegué et al. and Santaguida et al., in which JunB inactivation specifically expanded the number of long-term hematopoietic stem cells (LT-HDCs) leading to myeloproliferative disease [8,9]. Since the growth-promoting effects of telomerase have been linked to TGFβ inhibition and the expression of the TGFβ effector gene JunB is decreased in mice that overexpress mTert, it is apparent that the same, or closely related, pathway as that of Beckwith–Wiedemann syndrome may be involved in this tumor model [1]. One way to unify the cancer pathways of mature and stem/early progenitors is to hypothesize that the common outcomes of the independent mutations that are prevalent in common adult cancers may converge in a final step of telomere attrition/dysfunction, which, similar to the crisis observed in in vitro culture, might trigger reactivation of telomerase or ALT which is indispensable for the indefinite survival of cancer cells. Instead, tumors of stem cell origin appear to be driven by pathways that directly impact the telomere maintenance mechanism/s.

This broad division between stem cell and differentiated tumors leaves room for a gray zone of immature/early post-stem cell tumors where cancer pathways have some resemblance to those of stem cells in that a crucial feature of them is to induce accelerated exit from the stem compartment (a feature shared by the Santaguida and Passegue tumor model) [8,9]. This may suggest interference with the telomere-maintenance-specific mechanism/s of stem cells. A clue to this oncogenic route may be provided by the differential use of secondary oncogenic targets in mature versus immature B-cell lymphomas when tumor development is induced by the same initial oncogenic event. For instance, Eμ-c-Myc transgenic mice develop B-cell lymphomas of both mature and immature origin. Nepal et al. [10] showed that 90% of mature B-cell lymphomas (B220^+^ sIg^+^) overexpressed Mdm2, Arf, or p53 either alone or in combination, whereas none of the evaluated immature lymphomas in Eμ-c-Myc mice overexpressed Arf or p53 and only 27% overexpressed Mdm2. Western blot analysis of the c-myc-induced apoptotic pathway in the immature B-cell lymphomas revealed that none of the six evaluated tumors analyzed showed accumulation of Arf, Mdm2, or p53. These data indicate that c-myc induces the Arf-p53 pathway in mature B cells but not in pro- and pre-B cells [10]. Thus, although p53 activation is functional throughout B-cell development, its inactivation seems crucial only for mature, but not immature, tumors [10]. This is consistent with the concept of the two different functions of p53 at early and late phases of hematopoietic development. In fact, it has been reported that in the hematopoietic stem cell compartment, p53 regulates not apoptosis, but stem cell quiescence and exit from the stem cell pool through its targets Necdin and Gf-1 and independently of p21. The same concept is supported by specific knock-in p53 mutations that display different protective effects in mature and immature lymphomas [1]. Finally, it is interesting to point out that immature B-cell lymphomas display an accelerated onset compared to mature lymphomas. Another example of the use of different secondary oncogenic targets depending on the cell stage comes from the Ink4-ARF pathway: inactivating mutations in this pathway are probably not operative in stem cells because Ink4a-ARF is physiologically silenced due to the high expression of Bmi-1. However, at later stages, when Bmi-1 levels decrease and Ink4a-ARF is derepressed, loss-of-function mutation of this locus is expected to have a significant impact on growth deregulation [1]. For the same reason, tumors induced by any of these pathways (Ink4a-Arf, p53 apoptotic pathway in lymphomas, or c-Myc tumor model where c-Myc is expressed upon activation of the μ enhancer) cannot originate from stem cells. It has been shown that c-Myc overexpression within the stem cell compartment results in cellular exit from the stem cell compartment and increased differentiation at the expense of self-renewal [11]. Progenitors that leave the stem cell compartment as a result of c-myc overexpression become vulnerable to carcinogenesis. This may be associated with the impact of c-Myc on exit from the stem cell pool and probably interference with telomere maintenance mechanism/s specific to stem cells due to the ability of c-Myc to activate hTERT expression [12]. Information gathered on therapy using human-induced pluripotent stem cells (hiPSCs) revealed that tumorigenicity caused by reprogramming factors Oct3/4, Sox2, Klf4, and cMyc should be mainly attributed to cMyc reactivation. In Yamanaka’s words, “chimeric mice made with iPSCs created by the induction of retrovirus-mediated transfection of the four reprogramming factors often developed tumors”. We detected reactivation of c-Myc retrovirus in these tumors. Chimeric mice with iPSCs that had not been induced with the c-Myc retrovirus did not show such tumors. Another subset of tumors and teratomas formed by transplanted iPSCs may be related to faulty differentiation–expansion of progenitors in the absence of a physiological microenvironment, as shown by the fact that efficient methods of in vitro-directed differentiation reduce the risk of teratoma and tumor formation [13].

These concepts were developed in a previous paper (1) where a thorough examination of hematopoietic developmental blocks showed that they are always associated with subsequent cancer development [1]. The cancer stem cell of these tumors bears a phenotype corresponding to the stage of differentiation arrest caused by each specific block, a critical point in hematopoietic development associated with an anomalous DNA damage reaction. However, a causal explanation for these observations was not offered and was only loosely attributed to “replication stress”. Since developmental blocks, oncogenes, and other cellular insults such as radiation and reactive oxygen species make use of the same basic response apparatus, known as DNA damage response (DDR), which is used in genomic recombination events associated with cell differentiation, stalled replication forks (whose frequency should be higher in rapidly dividing cells such as transit amplifying cells), and other DNA insults, it is plausible that an impaired DDR may explain the more frequent origin of cancer in cells undergoing differentiation than in stem cells. This offers a simple explanation of this phenomenon. More significantly, it appears that a deregulated DNA damage response (whether associated with differentiation blocks or other genomic insults) can result in telomere dysfunction, thus closing the arguments in support of telomere dysfunction as the final triggering event in transformation of both committed and stem cells. In this view, cells in the stem cell compartment can only engage in malignant transformation through direct impairment of telomere maintenance as they are not usually exposed to the effects of impaired DNA damage responses. However, in committed cells, telomere damage may be the end result of the operation of a wide variety of DNA damage responses that occur along tissue turnover. In this view, anomalous telomere maintenance mechanisms brought about by aberrant DDR, rather than faulty repaired DNA, are responsible for malignant transformation since only telomeres can maintain indefinite self-renewal. Unrestrained cell proliferation induced by DNA mutation might also induce transformation by disrupting telomere maintenance.

Finally, some observations suggest that the interaction between oncogenic pathways and the telomere is a two-way road. Telomere dysfunction may also impact on genomic and cellular events necessary for survival of cancer cells independently of telomere maintenance.

## 2. Review and Hypothesis

### 2.1. The DNA Damage Response—Telomeres and Cancer

Genome damage can be caused by endogenous reactive molecules, environmental agents, or internal errors arising during the recombination of genomic regions as in the assembly of T- or B-cell receptors or other differentiation events as well as stalled replication and mismatch repair of DNA. Repair of genomic damage relies on two main DNA repair pathways. The first, homologous recombination (HR), occurs between the broken DNA and the corresponding region on the opposite strand and includes, among other components, Rad51, Rad52, Rad54, and c-Abl. The second pathway is nonhomologous end joining (NHEJ), which is carried out by DNA-dependent protein kinase (DNA-PK), its associated dimer Ku70–Ku80, and the DNA ligase IV/XCCR4 complex. Single (SSBs) and double-stranded breaks (DSBs) are the most lethal lesions of genomic DNA. Such DNA breaks may be produced as soon as cells exit the quiescent state of stem cells.

The response to DSBs is initiated by ATM and the MRN complex, whereas single-stranded breaks primarily activate ATR. ATM phosphorylates and activates downstream substrates such as Chk2, p53 at serine 15, and BRCA1 [14]. Many of these secondary targets are multifunctional proteins that interact with various repair pathways. This makes it difficult to dissect the interconnected processes involved in repair, checkpoint control, and telomere maintenance. For instance, Rad9 is phosphorylated by ATM and, upon binding of 9-1-1 and TOBP1, activates ATR, leading to Chk1 activation. Similar to p53, it may be involved in cell cycle arrest and apoptosis. Additionally, it participates in five DNA repair pathways: HR, base excision repair (BER), nucleotide excision repair (NER), mismatch repair, and alternative-NHEJ (alt-NHEJ) [15].

On the other hand, distinct DDR pathways may be partially redundant or require collaboration to achieve a single task such as cell cycle control. Chk2, phosphorylated by ATM, is responsible for the phosphorylation of p53 at Ser20, which is important for p53 stabilization because this modification interferes with the association with its inhibitor Mdm2, thus reinforcing the G1/S checkpoint. Chk1 (activated by ATR) and Chk2 act together to phosphorylate and inactivate phosphatase Cdc25C, which maintains the progress of the G2 phase by dephosphorylating and activating cyclin-dependent kinase Cdc2. Cdc25C phosphorylated at Ser216 creates a binding site for 14-3-3 protein and this complex is exported to the cytoplasm, thereby halting progression of G2 phase. Thus, ATR and ATM collaborate in cell cycle control. Downstream targets of ATM, ATR, and factors like cohesion can also mediate cell cycle control [16].

The aim of the DNA damage response is to preserve DNA integrity. This goal requires the assistance of a temporary arrest at different phases of the cell cycle (G1/S, S, and G2/M checkpoints). Faulty DNA repair may lead to apoptosis, although its effect may be neutral or result in mutations that enhance cell proliferation. Apoptosis is also a frequent outcome of faulty telomeric DNA repair; however, telomere damage could also result in the emergence of aberrant mechanisms of telomere maintenance. In particular, checkpoint defects, including those that deregulate mitosis, are more likely to affect telomeres. For instance, prolonged mitotic arrest has been shown to induce the formation of multiple damaged foci at telomeres [16]. It seems that the essential characteristic of cancer stem cells, indefinite self-renewal, can only arise through different forms of telomere damage, leading to the emergence of aberrant forms of telomere maintenance in committed cells. It is difficult to envision how increased cell proliferation, per se, could result in indefinite self-renewal. According to this hypothesis, DNA lesions cannot induce transformation by themselves but must go through a final step involving alteration of the telomere maintenance apparatus.

Perusal of the literature shows that deregulation of most DNA damage response components is concurrently associated with telomere dysfunction/erosion and cancer predisposition (Table 1 and Table 2).

Finally, deregulated expression of chromatin remodeling factors may also drive tumorigenesis and may be mediated by telomere dysfunction.

### 2.2. False Exceptions to the Correlation between Cancer–Telomere Dysfunction: Seckel Syndrome, Cockayne Syndrome, Trichothiodystrophy

In humans, Seckel syndrome type 1 is caused by an autosomal recessive mutation in ATR. Patients with this condition exhibit developmental defects but no cancer predisposition. Nevertheless, an autosomal dominant germline ATR missense mutation associated with oropharyngeal cancer was found in 24 individuals from a five-generation pedigree. ATR mutation segregated with the disease and resulted in lower p53 levels upon ATR activation. Loss of heterozygosity has been observed in tumor tissue [61]. Mice heterozygous for ATR develop normally and suffer increased cancer incidence after 18 months of age, while adult mice null for ATR displayed an age-related phenotype reminiscent of human Seckel syndrome without tumor development. ATR is specifically activated by single-strand DNA lesions. Initially, ssDNA intermediates are rapidly coated with replication protein A (RPA) which is quickly bound by ATRIP, providing a binding site for ATR. The DNA repair process that follows requires recognition of the junctions of the DNA lesion by Rad17 and the 9-1-1 complex and activation of several further components of the pathway, including TOBP1 and Fanconi Anemia factors. This suggests that DNA repair in Seckel syndrome is aborted in its initial stages, which may conceivably lead to arrested proliferation or be incompatible with cell life owing to ATR loss of function [62]. Dramatic proliferation failure of ATR-deficient cells is observed in vivo as well as in in vitro cell culture, apparently due to the lack of stabilization of replication forks in the absence or deficiency of ATR. Chromosome breaks are observed in ATR knockout cells even without exogenous replication stress, suggesting that stalled DNA replication may be a common event during normal replication. The rapid exhaustion of replication ability in ATR-deficient cells may explain the rarity of tumors either in ATR null mice or in human Seckel syndrome [63] or even the small occurrence of tumors in heterozygous mice suggesting that unrepaired DNA is incompatible with cell life, reinforcing the view that sustained cell proliferation must be supported by a putative anomalous telomere-associated mechanism rather than by genomic mutation; in other words, unrepaired DNA leads to cell death rather than cancer.

Cockayne syndrome (CS) is caused by a specific failure in the mechanism of transcription-coupled repair due to defects in either of the two genes ERCC6 (CSB) or ERCC8 (CSA). Following UV irradiation, transcription is arrested through the binding of ATF3, the product of an immediate early gene (IEG) to CRE/ATF binding sites present in numerous genes. This prevents binding of RNA pol II and inhibits RNA synthesis. Surprisingly, not only are transcribed genes inhibited by this mechanism but so are inactive genes. RNA sequence analysis showed that 5895 genes were downregulated (70%) in CSA and CSB cells. Inhibition of RNA synthesis is short-lived in cells proficient in CSA and CSB but considerably delayed in CS cells. ATF3 is removed from CRE/ATF sites at promoters by a ubiquitin proteasome degradation mechanism that involves Mdm2. Mutations that interfere with this process lead to the persistence of ATF and continued repression of RNA transcription similar to that caused by CSA or CSB deficiency [64]. Functionally, the end result appears to be analogous to that of Seckel syndrome in that interference with RNA transcription can make the cell unable to proliferate and execute DNA repair. As in the case of Seckel syndrome, this can be incompatible with cancer transformation.

A similar mechanism could explain the lack of cancer predisposition in trichothiodystrophy (TTD). TTD is caused by TTDN1, a gene of unknown function or by TTDA which encodes a small subunit of TFIIH. THIIH has a vital function in the initiation of RNA transcription [65].

### 2.3. Mutation of the Mre^11^ Component of the MRN Complex Does Not Result in Telomere Erosion nor Cancer Predisposition

The MRN complex formed by Mre^11^, Rad50, and Nbs1 proteins is the main sensor of double-strand breaks. It is involved in both the HR and NHEJ DDR pathways. MRN associates with chromatin and recruits inactive ATM dimers to DSBs where the active monomer initiates the ATM phosphorylation cascade. However, MRN or its components can also act downstream of ATM and, together with ATM, can promote the activation of ATR [14,66]. Germline alterations of MRN components give rise to hereditary cancer predisposition syndromes ataxia-telangiectasia like disease (A-TLD) and Nijmegen breakage syndrome (NBS), caused by hippomorphic mutation in the gene encoding Mre11 nuclease and NBS1 (encoding the protein NBN also known as nibrin) (null mutation of Nbs1 causes embryonic lethality). These syndromes are phenotypically similar to ataxia-telangiectasia, (AT), a hereditary cancer predisposition syndrome caused by ATM deficiency which presents with cerebellar degeneration, immunodeficiency, reduced fertility, radiosensitivity, and cancer predisposition. Similar phenotypic features are shared by Nijmegen breakage syndrome (NBS) except that it presents with microcephaly in place of cerebellar degeneration and A-TLD, although the latter may have a milder clinical expression [67,68]. Cancer predisposition of AT and NBS is conspicuous and manifests mainly in lymphomas related to chromosomal aberrations at the sites of T-cell receptor or immunoglobulin chain rearrangement where V(D)J recombination spontaneously induces DSBs. However, other cancer types may occur. Tauchi et al. [69] reported that 22 out of 55 patients in the International NBS registry in 2002 (age range 1–22 years) had developed a malignancy. Of these, 16 were lymphomas and the remaining six had developed leukemia, glioma, medulloblastoma, or rhabdomyosarcoma. Similar to AT, cells derived from patients with NBS exhibit accelerated shortening of telomeres, loss of intra-S and G2/M checkpoint control, and genomic instability [69]. Another study including 241 cases reported a median age of cancer onset of 9.1 years with a probability of 20-year survival of 44.6% [70], whereas a murine model in which exons 4 and 5 were replaced by neo did not show increased tumor incidence [71]. Dumon-Jones et al. generated heterozygous knockout (Nbn^+/−^) mice that developed a wide array of tumors in addition to lymphomas and gamma-radiation-enhanced tumor development [72]. Apoptosis does not seem to have an important role in tumor prevention in NBS. Thierfelder et al. found a high variation in apoptotic capacity in 30 lymphoblastoid cell lines from 30 patients, 17 of which belonged to patients who had developed cancer. The level of apoptosis did not correlate with the cancer incidence [73]. Although there are discrepant findings regarding tumor incidence in animal models of NBS, the occurrence of malignancy among NBS heterozygotes may be even higher than that in AT heterozygotes patients [74].

The association of cancer with A-TLD is more ambiguous, partly due to the rarity of this syndrome, although it is clearly much lower than that of AT and NBS. By 2020, only 23 cases had been identified, but only two patients died of cancer (lung adenocarcinoma) at 9 and 14 years of age. These were two brothers with two distinct mutant alleles, one from each parent.

A study by Barkova et al. comprising 1000 breast carcinomas showed reduced expression of the NBN protein (nibrin) in (10% of cases) followed by Mre11 (7%) and Rad50 (3%). Sequence analysis identified two putative sequence variants of Mre11. Neither mutation was found in 332 familial breast cancer patients. The missense mutation was present in 1/363 population controls while the stop mutation was not found in controls. However, the missense mutation was in its heterozygous state on stromal cells while tumor cells had lost the second. There was a trend for higher presence of mutations of MTN complex in association with BRCA mutations. This study confirmed the well-known fact that mutations in Mre11 are associated with the reduction or loss of other proteins of the MRN complex, which makes the specific role of Mre11 in cancer predisposition even more elusive [75]. However, the occurrence of germline Mre11 alterations in a minority of breast cancer patients observed in these studies has not been confirmed in ample case population studies [70]. It has also been suggested that mutations in Mre11 are associated with increased susceptibility to colorectal cancer. However, these mutations have been found exclusively in the context of mismatch-repair deficiencies [76].

Gupta et al. [77] addressed the role of Mre11 in the oncogene-driven DNA damage response. Expression of activated NeuT in the murine ductal mammary epithelium across different genotypes induced epithelial hyperplasia 3 weeks post-injection, which correlated with oncogene expression. Mre11^ATLD/ATLD^ and NBS1^∆B/∆B^ developed more florid hyperplasia which appears to be associated with defects in the intra-S and G2/M checkpoints. Gland hyperplasia in wt NeuT-injected cells progressed to mammary tumors in 5% of gland within a 6-month period. In contrast, 40% of the hyperplastic glands from Mre11^ATLD/ATLD^ developed tumors during the same period. Chk2^−/−^ and Nbs1^∆C/∆C^ Chk2^−/−^ mice developed tumors at a rate indistinguishable from that of wt. Increased tumorigenesis in the Mre11 mutant may reflect the more pronounced chromosome fragility and apoptotic defects observed in this genotype. Indeed, metaphase analysis demonstrated a sevenfold increase in the rate of spontaneous chromosomal aberrations relative to the wt. The Neu-driven DDR mediated by Mre11 induces accumulation of cells in late G2, as demonstrated by the deposition of phospho-histone H3 serine 10 (pHH3-S10) at pericentromeric heterochromatin. There was a fourfold increase in the deposition of pHH3-S10in WT relative to Mre11^ATLD/ATLD^, showing that cells accumulate at G2 in normal cells but less so in Mre11 mutant cells. However, the final cancer transformation appears to be dependent on the concomitant alteration of the Ink4a/Arf gene, which is favored in Mre11 mutants. There was an initial increase in the expression of p19Arf in Mre11^ATLD/ATLD^ followed by deletion of exon 2 of Ink4a which disrupts both the Ink4a and p19 transcripts. As expected in the NeuT-driven model, mammary tumors were of the basal type. A further observation that deserves to be noted is that the oncogene or Neu-driven DDR response showed some features different to those of an IR-induced DDR.

This experiment showed that checkpoint control afforded by intact Mre11 may contribute to the suppression of oncogene-induced tumorigenesis.

However, other reports have revealed some oddities regarding the suppressive action of Mre11 [78]. In one such experiment, researchers used Mre11^H129N^ mice deficient in nuclease activity. Nuclease activity is dispensable for ATM activation. The Mre11^H129N^ mutation was introduced into B lymphocytes through CD19-Cre expression. Upon Cre expression, Mre11^cond/+^ Mre11^cond/−^ and Mre11^cond/H129N^ became Mre11^−/+^ (control) Mre11^−/−^ and Mre11^−/H129N^, respectively (cond refer to the LoxP sites that allow elimination of the gene). It may be useful to describe these experiments in detail:

After introduction of the Mre11^H129N^ mutation in B lymphocytes through CD19-Cre expression, Mre11^cond/+^ Mre11^cond/−^ and Mre11^cond/H129N^ gave rise to Mre11^−/+^ (control cells) Mre11^−/−^ and Mre11^−/H129N^, respectively. Survival of P53null Mre11^−/−^ and p53 null Mre11^H129N/−^ (16–18 weeks) was similar to control p53 null Mre11^+/−^ (16–18 weeks). Necropsy found that the majority contained thymic lymphomas typical of p53 deficiency. Therefore, it appears that B-lymphocyte-specific deficiency of Mre11 or Mre11 nuclease activity does not affect the survival of mice.

Next, they examined the impact of Mre11 deficiency on the Artemis mouse model strongly predisposed to B lineage lymphomas. Artemis is required for processing the DNA ends generated during V(D)J recombination. Mutations in Artemis cause immunodeficiencies and tumors. When combined with p53 deficiency, aggressive pro-B lymphomas harboring chromosomal translocations involving IgH and c-Myc or N-Myc loci develop. The results were as follows:

Artemis null, p53 null, Mre11 null (17 weeks survival).

Artemis null, p53 null Mre11^H129n/−^ (19 weeks survival).

Artemis null p53null Mre11^+/−^ → (14 weeks survival).

Thus, the B-cell-specific loss of Mre11 or Mre11 nuclease activity did not markedly alter survival. In fact, survival was slightly prolonged in Mre11 null or ^Mre11/−^. The majority of Artemis/p53 double nulls succumbed to progenitor B lymphoma and a smaller subset of thymic lymphoma of progenitor T-cell origin. Remarkably, no pro-B-cell lymphoma arose in Artemis/p53 double null mice with either Mre11^−/−^ (*n* = 11) or Mre11^−/H129N^. These results indicate that the Mre11 mutation suppresses pro-B-cell lymphomas in a context where pro-B-cell lymphomas normally arise in the majority of mice by 8–10 weeks of age in spite of the preserved ability of p53 null Mre11 null and p53 null Mre11^H129N/−^ to generate oncogenic chromosomal translocations (including IgH: Myc translocations) and also chromosomal translocations outside of the IgH locus, not only in B cells but also in nonlymphoid cells. In summary, inhibition of Mre11 nuclease activity through gene mutation precludes or attenuates tumor promotion.

Thus, the cancer predisposing role of Mre11^ATLD1/ATLD1^ occasionally reported appears to be very slight.

This is supported by the work of Theunissen et al. [79] using the Mre11^ATLD/ATLD^ mutant. They showed that Mre11^ATLD/ATLD^ mice are not prone to lymphomagenesis in spite of having defective cell cycle checkpoints and chromosomal translocations similar to that of ATM and NSB1 deficiencies, whereas the apoptosis rate was only slightly reduced in comparison to w.t. Tumor latency was modestly reduced in the background of p53 deficiency, although the modest difference observed in the age of death should not be entirely attributed to increased malignancy of the double Mre, p53 mutants as many of them did not exhibit overt malignancy upon necropsy.

A feature displayed by the Mre11^ATLD/ATLD^ mutation might explain the different tumor-promoting activities of MRN component Mre11 versus Nbs1 and ATM. Shortened telomeres associate with defective ATM or Nbs1 function [69], but telomere shortening is absent in cells homozygous for Mre11^ATLD/ATLD^ [80]. Attwool et al. reported that no telomere attrition was seen in immortalized Mre11^ATLD/ATLD^ MEFs over the course of over 300 population doublings. At cPDL 300, there was no difference in telomere shortening between Mre11^+/+^ Tert^∆/∆^ and Mre11^ATLD/ATLD^ Tert^∆/∆^. Also, maintenance of the G-strand overhang was unaffected. The rate of telomere fusion was reduced in Mre11^ATLD/ATLD^ Tert^∆/∆^ (5% of all chromosomes fused compared to 8% in Tert^∆/∆^). Fewer fusions were also detected in Mre11^ATLD/ATLD^ Tert^∆/∆^ than in Mre11^+/+^ Tert^∆/∆^ [80].

In summary, although DNA repair, checkpoint control, genomic stability, and telomere maintenance appear to be disrupted simultaneously by mutations of ATM or Nbs1, and are associated with their cancer predisposition, mutation of the MRN component Mre11 (Mre11^ATLD/ATLD^) does not impair telomere integrity despite affecting other branches of the DDR. Concomitantly, this mutation is associated with a much smaller cancer predisposition than that associated with ATM or NBS1, suggesting that telomere disruption is the main culprit for the ensuing cancer predisposition (Table 3).

Mutations in Rad50, the other subunit of the MRN complex, are very rare. To date, only two patients with germline biallelic variants of Rad50 have been described. The clinical presentation was similar to both NBS and A-TLD. The association of either homozygous or heterozygous variants of Rad50 with cancer predisposition has been debated [81]. However, Rad50^s/s^ or BRCA1 defects giving rise to progeroid phenotypes, shortened lifespan, cancer predisposition, and hematopoietic stem cell failure has been described [82].

### 2.4. Chromatin Remodeling Factors Are Concurrently Associated with Telomere Dysfunction and Cancer

T-loops are RNA: DNA hybrids formed between a single RNA strand and a double-stranded DNA. Telomeric repeat containing RNA (TERRA) is transcribed from subtelomeric regions to telomeres and can invade telomeric dsDNA to form telomeric R-loop structures [83]. The SWI/SNF chromatin remodeling complex plays an important role in resolving R-loop conflicts. Consequently, malfunction of this complex may result in telomere dysfunction. Deregulation of SWI/SNF has been observed in undifferentiated/rhabdoid-type tumors [84,85]. This suggests that its normal function must occur at a very early stage of cell differentiation and its impairment must be reflected in the very immature phenotype of the tumor cell population.

BCL11B is a transcription factor that interacts with nucleosome remodeling and the histone deacetylation complex NuRD [86]. It may also play a role in telomere structure and function [87,88]. Mutations in BCL11B have been associated with lineage ambiguous leukemias [89] and undifferentiated nervous tissue tumors [90]. There are several other cases where chromatin remodeling dysfunction is associated with acute leukemias and sarcomas with an extremely undifferentiated phenotype, suggesting that these tumors arise through errors produced in very early differentiation events at the immediate post-stem cell stage.

Another member of the SWI/SNF chromatin remodeler group, ATRX, plays an important role in telomere function and cancer which is beginning to be unraveled. Loss of ATRX or its partner DAXX frequently occurs in cancers with the ALT phenotype. It has been shown that the loss of these factors induces telomere dysfunction that culminates in the inception of an ALT mechanism that supports cell immortalization [91].

### 2.5. Possible Routes of ATM–Telomere Interaction

One of the substrates of ATM that is activated by DNA damage is Pin2/TRF1. ATM phosphorylates and inhibits Pin2/TRF1. Pin2/TRF1 interacts with the telomerase inhibitor PinX1. A lower level of endogenous Pin2/TRF1, induced by the expression of dominant negative (DN) mutants in A-T cells or phosphorylation of Pin2/TRF1 on Ser219 by ATM following DNA damage, prevents telomerase inhibition by PinX1, leading to telomere elongation. Conversely, higher expression of Pin2/TRF1 or Pin2/TRF1 mutations refractory to Ser219 phosphorylation results in phenotypes similar to those of ATM mutations, including telomere loss and accelerated mitotic entry due to impaired G2/M checkpoint (the S phase checkpoint is not affected by Pin2/TRF1) [92].

Pin2/TRF1 overexpression induces mitotic entry and apoptosis. Upon DNA damage, ATM phosphorylates Pin2/TRF1, thereby inhibiting its interaction with microtubules and preventing apoptosis and mitotic entry. This is achieved through the ATM-dependent phosphorylation of Pin2/TRF1 on Ser219. In this way, cells can delay entry into mitosis and apoptosis when experiencing ionizing radiation and ATM activation.

One of the substrates of Pin2/TRF1 is the telomerase inhibitor PinX1 [93]. This protein is localized at the nucleolus and at telomeres. PinX1 inhibits telomerase activity, and depletion of endogenous PinX1 increases telomerase activity of telomerase-positive cells and increases tumorigenicity. It is unclear whether ATM-dependent Ser219 phosphorylation is also responsible for altering the interaction of Pin2/TRF1 with PinX1 and thereby suppressing PinX1 telomerase inhibitory activity. At any rate, dominant negative mutant Pin2/TRF1, mutation of Ser219 to A219, direct inhibition of Pin2/TRF1, or ectopic expression of yeast Tel1 (the yeast orthologue of Atm) rescued both telomeres shortening and the G2/M checkpoint defect, presumably via phosphorylation. However, yeast tel1 does not rescue the S checkpoint defect, in contrast to Atm.

Interestingly, Pin2/TRF1 increases during late G2 and M stages and its ability to induce mitosis and apoptosis depends on telomere length. Apparently, in cells with long telomeres Pin2/TRF1 remains largely bound to telomeres, whereas it is free in cells with short telomeres [94]. This suggests the possibility that a normal ATM response in a cell with short telomeres and Pin2/TRF1 outside telomeres could result in telomerase expression unrelated to telomere elongation (on the assumption that telomere elongation could only be accomplished through lack of telomerase inhibition of Pin2/TRF1 at telomeres). Thus, Pin2/TRF1 inhibition by ATM could result in telomerase functions unrelated to telomere length preservation, i.e., oncogenesis as well as delayed mitotic entry. However, ATM deficiency should result in telomere shortening in cells with long telomeres.

The important role played by PinX1 in oncogenesis can be estimated by a study by Huang et al., where PinX1 gene alterations were determined in 105 separate studies using the online resource cBioportal. Four alterations (mutation, deletion, amplification, and multiple alterations) were detected in 53 studies, with Pin deletions accounting for most alterations followed by mutations in the PinX1 functional domain (nucleolar localization domain and telomerase inhibitor domain). PinX1 deletion was frequently associated with carcinosarcoma and adenocarcinoma [95].

Simultaneously, ATM disturbance (as well as ATR) impairs cell cycle control [96], which might deregulate telomere maintenance as well as other late-replicating and DNA regions rich in repetitive elements (see below).

### 2.6. Double-Stranded Breaks versus Chromatin Alteration

The role of physiological DSBs and their alterations in tumorigenesis was studied using a different model by Petiniot et al. [97]. These authors showed that mice double deficient for the recombinase-activating gene Rag 2 and Atm were still subject, in the absence of physiological V(D)J recombination, to increased tumor development both in lymphoid tissue and other tissues. It is known that lymphomas arising in ATM-deficient mice display consistent cytogenetic abnormalities at the Tcr/α/δ locus. The work of these authors demonstrated that development of malignant lymphoma in Atm^−/−^ mice is not prevented by loss of Rag2. However, the appearance of these tumors was somewhat delayed, and lymphomas did not exhibit the Tcr/α/δ rearrangement typical of ATM deficiency. In order to evaluate the role played by developmental arrest produced as a result of lack of Tcr, these authors generated HyTcrTg^+^ Rag2^−/−^ Atm^−/−^mice that overcome arrest by HyTcr transgene expression. These mice did develop lymphoma comparable in frequency and phenotype to those of Rag^−/−^ Atm^−/−^, and cytogenetic analysis demonstrated absence of translocation within the Tcrα/δ locus.

As expected, no tumors developing in Rag2^−/−^ Atm^−/−^ mice express surface TCR. However, when the arrest in T-cell development caused by Rag2 deficiency is rescued by TCR transgene expression, as in HyTcrTg^+^ Rag2^−/−^ Atm^−/−^ [98], all tumors expressed the TCR transgen V_β_8^+^. The predominance of tumors expressing the transgene might suggest that the proliferation burst provided by TCR expression may contribute to tumor development. Nevertheless, the ability to proliferate and differentiate must not be completely abolished in the absence of TCR formation, as shown by surface expression of CD4 and CD8. In fact, some tumors developing in Rag2 null mice were, as expected, CD4- and CD8-negative, but others expressed varying levels of these differentiation markers. Furthermore, a subpopulation of CD8+ cells was found in one CD4^−^ CD8^−^ tumor. These results suggest that the role of Rag2 in thymocyte differentiation may be bypassed in some cases, as shown by the previous finding that CD3^−^ CD4^+^ CD8^+^ thymic lymphomas are found in Rag1^−/−^ p53^−/−^ and Rag2^−/−^ p53^−/−^ mice [99].

For unknown reasons, nonmalignant peripheral T cells from ATM-deficient mice harbor a greater than 20-fold increase in chromosome 14 structural abnormalities as compared with peripheral T cells from wild-type mice. Thus, as expected, tumors from both HyTcrTg^+^ Rag2^−/−^ Atm^−/−^ and Rag2^−/−^Atm^−/−^ exhibited a translocation involving either chromosome 14 or chromosome 12, both of which are homologous to human chromosome 14. This bias was attributed to a more open conformation of chromatin structure necessary for V(D)J recombination [97]. These results suggest that the initial event in transformation is a sporadic or stochastic DNA initiating lesion, perhaps not necessarily a DSB, which is not efficiently handled due to ATM deficiency. The examples described above suggest that tumorigenesis in ATM deficiency is dependent on abnormal responses to DNA breaks rather than defects in V(D)J recombination.

## 3. Replication Stress Associated with Developmental Blocks Underlies Cancer Transformation

Examination of hematopoietic development showed that any developmental block at any step of the differentiation ladder is always associated with leukemic transformation [1]. On this basis, it was proposed that replication stress induced by blocked differentiation induced transformation by disrupting telomere integrity and/or the re-expression of telomerase [1]. Developmental blocks create favorable conditions for DNA damage by altering chromatin structure, delaying or stalling transcription, and generating recombination breaks that, when not properly handled, can be converted to single- and double-stranded DNA breaks. Moreover, it has been shown that cells unable to transition to the next differentiation step proliferate extensively, and, consequently, a local shortage of nucleotides may be generated, an important feature of replication stress [1].

### 3.1. Replication Stress

Replication stress (RS) refers to disturbances in DNA dynamics that are reflected in the slowing and asymmetry of replication fork progression. It also affects deregulation of replication origins. There are multiple causes of replication stress, including oncogenes, shortage of nucleotides and replication factors, UV or ionizing radiation, and reactive oxygen species, as well as difficulties arising in the process of DNA replication due to special features of particular DNA regions, such as repetitive elements late replication or anomalous chromatin configuration. Some of these causes have been shown to be interrelated, such as oncogenes and exhaustion of nucleotide pools that preferentially influence certain regions of DNA such as common fragile sites (CFS). Concerning oncogenes, Bester et al. showed [100] that keratinocyte proliferation induced by deregulation of the Rb-E2F pathway by HPV-16 E6/E7 or cyclin E oncogenes resulted in slower cellular DNA replication fork rate, decreased symmetry between the right and left fork progression, and increased number of active origins. They detected increased copy number variation (CNV) and loss of heterozygosity (LOH) preferentially associated with fragile sites. LOH measured after 100 days in three representative fragile sites (FRA3B, FRA16D, and FRA7G) was 4.1% significantly higher than that found in the entire genome, 1.4%. Instability increased with time. It was firmly established that both replication slowing and DNA damage were caused by an insufficient rate of nucleoside synthesis which was unable to cope for the needs of enhanced cell proliferation induced by the Rb-E2F pathway. In fact, exogenous supply of nucleosides corrected the DNA-replication-induced defects without affecting cell proliferation. The main form of DNA damage detected was activation of the ATR DDR pathway caused by the slowing of replication forks [100].

Saldivar et al. [101] demonstrated that deletion of the FHIT gene located within the FRA3B fragile site results in nucleotide exhaustion because FHIT knockdown suppresses thymidine kinase 1 (TK1) expression. The primary DNA defect observed was deficient replication fork progression which led to fork stalling and collapse. FIHT knockdown resulted in a threefold increase in the fraction of cells containing phosphorylated ATR and γH2AX foci but they did not detect phosphorylated Chk1, suggesting that the S phase checkpoint was not activated. In fact, S and G2 checkpoints were defective and cell proliferation continued. This suggests that it is the branch of the DDR responsible for delaying cell division that is mainly perturbed. Loss of FHIT is frequent in precancerous lesions and has been proposed as a soil for further cancer transformation [92]. Similarly, hydroxyurea (HU) causes depletion of dNTPs through inhibition of ribonucleotide reductase (RNR), and replication fork stalling is typically observed after treatment with hydroxiurea (HU) or aphidicoline. Regulation of dNTP production is considered an essential branch of DNA damage signaling but has been mainly studied in yeast. In mammalian cells it has been shown that ATR may promote stabilization of RRM2, the regulatory subunit of RNR [102].

Similar to common fragile sites, other loci such as telomeres, centromeres, and ribosomal DNA are especially prone to replication stress due to their rich content in DNA repetitive sequences, special chromatin configuration, or late replication. Here, replication stress is frequently reflected on transcription–replication conflict (TRC) and formation of R-loops (DNA–RNA hybrids and looped-out single stretches of DNA). These structures can undergo breakage if not properly resolved, particularly during mitosis.

A novel function displayed by some members of the FA pathway interacting with BRCA proteins, and distinct from its canonical function in homologous recombination (HR)-mediated repair, seems to be critical for maintenance of chromosomal stability during replication stress. FANCD2 is localized to distinct spots on the mitotic chromosomes. These spots were more numerous after replication stress induced by APH and formed structures intimately linked to these DNA sites that results from sister chromatid entanglement known as ultrafine bridges (UFBs). There are five types of ultrafine bridges arising from each of these sites: centromeric (c-UFBs), ribosomal (r-UFBs), common fragile site (CFB-UFBs), telomeric (t-UFBs), and the newly identified HR-UFBs derived from HR recombination intermediates. Most of this research has been conducted on CFSs; however, it appears to be equally applicable to t-UFBs.

It has been shown that higher levels of transcription–replication conflict can activate ATM even in the absence of DSBs. For instance, after HU treatment, the helicase FBH1 has been shown to activate ATM in response to fork stalling independently of DSB, and depletion of FBH1 helicase results in accelerated mitotic entry. Collaboration between ATM and p53 in mitotic control has been observed in nontransformed cells. ATM-induced phosphorylation of p53 results in centrosome localization of p53 in mitotic centrosomes, whereas impairment of this process results in cell death ([103] and references therein). ATM activation could also be deleterious to telomeres due to its role in telomere attrition.

Tolerance to low levels of replication stress or defects in checkpoint and mitotic control resulting from deficient resolution of chromatid entanglement can easily lead to the loss of DNA sequences. In CFSs, this may underlie the frequent inactivation of tumor suppressors in these regions while in telomeres it will likely be reflected by changes in length and/or telomere dysfunction, whereas loss of centromeric or ribosomal DNA will most likely lead to impaired cell function or even cell death. We hypothesize that loss of tumor suppressors or mutations in genomic DNA can enhance cell proliferation, which cannot result in transformation unless a new telomere maintenance mechanism arises. Telomere dysfunction can also result in cell arrest or cell death, but in some cases may lead to escape of cell death through different mechanisms that maintain telomere length and continuous cell division. It is not easily envisioned how this outcome may result from signaling from other DNA regions.

### 3.2. Paradoxical Effects of Chk1

Replication stress caused by agents such as thymidine or hydroxyurea has been found to be associated with decreased level of ribonucleotide reductase and, apparently, activation of Chk1, which may suppress the firing of new replication origins and slow replication fork progression. However, depletion of Chk1 by small interfering RNA in the presence of these RS-inducing agents results in a higher level of apoptosis [104]. Conversely, transgenic mice containing an extra copy of Chk1 show a diminished RS response, as detected by a reduced number of ssDNA-binding protein RPA foci after treatment with RS-inducing agents. In addition, the extra Chk1 copy prolonged survival in the ATR-Seckel mice model which suffer from high level of RS. Cells from these mice presented a hyperactive IR-induced G2/M checkpoint. Paradoxically, however, the increased levels of this tumor suppressor favored transformation [105]. Presumably, faulty telomere repair will be associated with slower replication fork progression and delayed repair of genomic DNA.

### 3.3. Other Roles of ATR and the Fanconi Anemia Pathway

Efficient activation of ATR signaling and remodeling of stalled replication forks requires the Fanconi Anemia pathway (FA). The FA pathway is thought to comprise 22 genes organized into three groups. Group I consists of eight proteins that form the FA core plus associated proteins FANCM-FAAP24, FAP20, and FAP100. The primary function of this pathway is the protection of DNA replication forks and repair of interstrand cross-links (ICLs). Single-strand and double-strand breaks are subproducts of ICL processing. FANCM can recognize ICLs during S phase and recruits the FA core complex [106] which leads to monoubiquitination of the FANCD2-FANCI (group II or ID2) and further recruitment of group III proteins that mediate different aspects of the DNA repair function such as DNA incision, ICL elimination, translesion synthesis, homologous recombination, etc. [97]. The FA pathway has been shown to be important for protection of specific regions of the genome called common fragile sites, where large genes, including tumor suppressors, are located [107]. However, telomeres may be the most fragile of all fragile sites. FANCM depletion led to reduction of DNA replication especially severe at telomeres (reduction at whole genome DNA was 1.4-fold versus threefold at telomeres). Another interesting aspect of FANCM and FAPP24 proteins is their ability to bind HLCK2 independently of the FA core complex [108]. The HCLK2/Tel2 gene, originally identified in C. elegans, is one of the most enigmatic players in the DNA damage response. There is no evidence that its higher eukaryote ortholog can bind DNA. Nevertheless, it has been shown to be involved in DNA damage and checkpoint control. Furthermore, HCLK2 function seems to greatly influence the balance between DNA damage/repair and checkpoint signaling. Horejsi et al. showed that a partial reduction in ATR levels resulted in a severely compromised checkpoint response [109]. This unbalanced situation, which, as mentioned above, is associated with or can precede malignant transformation, might be communicated to the telomere complex either through an HCLK2/Tel2 downstream target or through HLCK2 itself. This function of HCLK2/Tel2 could be supported by its ability to differentially regulate members of the PIKK family but more likely would rely on a capacity, that has been demonstrated in its yeast homologue Tel2, called the telomere position effect, which enables this gene to control telomere length and silencing of genes in the subtelomeric regions [110]. Disruption of the telomere position effect may influence distinct functions of the telomere complex such as hTERT expression [111] and, in turn, aberrant TERT expression may initiate transformation. The ability of HCLK2/Tel2 to transmit the initial activation of DNA damage from ATR to the telomere provides a hypothetical common mechanism that may underlie oncogenic signaling in both familial cancer syndromes such as Fanconi’s anemia and sporadic cancers. A link between a perturbed DNA damage response and the telomere oncogenic pathway could be mediated by ATR-HCLK2/Tel2-Telomere signaling.

## 4. DNA Damage Associated with Defective Genomic Recombination

### 4.1. Oncogenesis and Altered Differentiation to Plasma Cells

Sherman et al. [112] analyzed a single differentiation event in hematopoietic development: the transition from germinal center lymphocytes to memory B cells and plasma cells. Their work showed that the impairment of normal differentiation can lead to neoplastic transformation in a process that recapitulates most of the features involved in developmental blocks. This analysis also showed that aberrant DDR is functionally equivalent to a differentiation block.

In the germinal center (GC) reaction, physiological double-strand breaks are produced during the diversification of antigen receptor genes and the formation of higher affinity antibodies associated with class switch recombination. They showed that, following a double-strand break (DSB), one of the substrates phosphorylated and activated by ataxia-telangiectasia-mutated (ATM) is LKB1 (also called STK11 (serine-threoninkinase 11), which, through adenosine-monophosphate-activated protein kinase (AMPK), induces cytoplasmic translocation and inactivation of CREB-regulated transcriptional coactivator 2 (CRTC2). This occurs over an interval of 3 to 7 days, correlating with a gradual decrease in the association between CRTC2 and the TCL1 promoter followed by concomitant gradual decrease in the expression of T-cell leukemia/lymphoma 1 (TCL1) oncogene. During the first phase of the GC reaction, B lymphocytes undergo enhanced proliferation due to TCL1 oncogene and BCL6 expression. B-cell lymphoma 6 (BCL6) imposes a pause in plasma cell differentiation through repression of the PR domain zinc finger protein 1 (PRDM1) (encoding B-lymphocyte-induced maturation protein 1 (BLIMP-1)) and suppresses some components of the DNA damage reaction (DDR) including ataxia-telangiectasis and Rad3-related (ATR) p53 and p21. This allows unrestricted B-cell proliferation, which must be inhibited in the second phase of the process. This requires BCL6 downregulation to allow for the next final differentiation step into plasma cells. The pathway in charge of this second phase, which abates the initial cell proliferation and permits differentiation, is also triggered by the physiologically generated double-strand break, in this case through activation of the ATM cascade, which antagonizes the repression of differentiation induced by BCL6. In addition to classical substrates activated by ATM, such as p53 and p21, these authors identified the tumor suppressor LKB1, which inactivates CRTC2. The timing of cytoplasmic translocation and inactivation of CRTC2 appears to be critical for the development of two opposing phases that entail delayed plasma cell differentiation in the initial GC reaction and proper differentiation and halted proliferation in the second phase, as reproduced in an in vitro B-cell differentiation system using naïve human tonsil B cells. In summary, the response to physiological DSBs is chronologically regulated and involves a first phase of repressed differentiation and enhanced proliferation, which is subsequently antagonized by mechanisms that inhibit proliferation and allow cell differentiation. The normal function of the DDR underlies the normal course of this process, and disruption of the second controlling phase (either by cell cycle arrest or apoptosis) has potentially damaging consequences that may lead to cancer development. The TCL1 oncogene expression associated with the proliferative phase of the GC reaction stimulates cell proliferation, apparently through its ability to hyperactivate AKT, and its downregulation coincides with gradual CRTC2 cytoplasmic translocation and inactivation between 3 and 7 days following class switch recombination (both BCL6 downregulation and CRTC2 inactivation appear to be necessary for plasma cell differentiation). Maintenance of TCL1 expression was observed in 11 of 13 B-cell lymphomas harboring deficiencies of the ATM-LKB1-AMPK-CRTC2 signaling pathway.

These experiments suggest an oncogenic pathway that could be extrapolated to leukemias/lymphomas that arise after hematopoietic developmental blocks that, like class switch recombination, are generally associated with DNA double-strand breaks. The identity and genomic lesion responsible for each developmental block should vary in accordance with the altered factor responsible for each differentiation step, but the mechanism of transformation seems to involve the same inability to transition to the subsequent differentiation stage. As in a GC reaction, disruption of the mechanism that stops cell proliferation, thereby halting cell differentiation, is likely involved. Targets involved in cell proliferation may vary according to the phenotype of the differentiation stage. For instance, TCL1 expression, which is observed from pre-B cells to mature lymphocytes, is aberrantly maintained in plasma cell tumors but is probably not involved in the early stages of development. However, DNA recombination that violates the normal differentiation program may also lead also to disturbed DDR, deficient ATM signaling, impaired differentiation, and unrestricted cell proliferation. Physiological DNA damage generated by the recombinase activating gene (RAG) during class switch recombination (CSR) requires ATM, whereas ATM knockdown results in nuclear localization and activation of CRTC2. This study suggests that aberrant and physiological DNA damage share common signaling circuits and therefore similar mechanism of cancer transformation [112].

### 4.2. Tumorigenesis Associated with Class Switch Recombination and Somatic Hypermutation Induced by Activation-Induced Cytidine Deaminase (AID)

Activation-induced cytidine deaminase (AID) is specifically expressed in germinal center B cells and has been shown to be required for somatic hypermutation, the process that introduces point mutations that diversify the Ig variable region during antigen stimulation as well as in class switch recombination (CSR). It is thought to play a role in kataegis and chromosomal translocations, especially those that cannot be explained by V(D)J recombination. AID may bind to G-loops formed within the transcribed c-myc gene, and the c-myc promoter is essential for recruiting AID and inducing DSB followed by c-Myc/IgH translocations. The physiological role of AID is the conversion of cytidine to uracil, which is necessary for somatic hypermutation and CSR. It has been speculated that AID can promote carcinogenesis by increasing the mutation load [113]. However, constitutive expression of AID was followed by T-cell lymphomas and dysgenetic lesions in the epithelium of respiratory bronchioles, rather than GC lymphomas. Thus, AID carcinogenesis appears to be associated with off-target genomic effects, which could conceivably induce defective DNA damage responses [114].

### 4.3. A DNA Damage Response Is Triggered by Oncogenes

A two-phase response similar to the germinal center (GC) reaction is also elicited by c-Myc. It differs from the GC reaction in the identity of the regulators driving the proliferative phase which, in this case, is directly induced by Myc and its targets, whereas the following growth suppression phase is triggered at least in part, as in the GC reaction, by a DDR response. As a matter of fact, in addition to signaling to p53 through the ARF tumor suppressor, enhanced Myc expression was shown to induce increased levels of total and phosphorylated p53 through ATM activation as part of a DDR response. Overexpression of Myc in squamous epithelium using K5-Myc transgenic mice demonstrated that Myc induced multiple γH2AX and phospho-SMC1 foci similar to those induced by ionizing radiation (IR) as well as malignant transformation.

It has been reported that forced expression of other oncogenes, including E2F1, cyclin E, or cdc25A, induce the ATM signaling pathway [115].

### 4.4. Disturbed Differentiation and Notch Signaling

The lack of coordination between physiological DNA damage and the subsequent phases in command of proliferation and/or mitosis control has been studied in an experimental model commented in [1] describing the association between hematopoietic developmental blocks and leukemogenesis:

Notch1 signaling controls the early development of progenitors into the T-cell lineage while inhibiting B-cell differentiation. Early thymic progenitors (ETPs) with the phenotype CD44^+^ CD25^−^ (DN1 stage) give rise to dendritic cells, NK cells, and T lineage committed CD44 + CD25+ cells (DN2 stage). Lineage commitment is followed by VβDβJβ recombination which, in combination with a surrogate light chain and other molecules such as CD3 chains, forms the pre-T-cell receptor (pre-TCR) complex. Completion of the pre-TCR divides the DN3 stage into DN3a and DN3b stages. Pre-TCR signaling is required for the differentiation of CD44^−^ CD25^+^ (DN3) into DN4 and double-positive (DP) cells. Disruption of pre-TCR arrests T-cell development at the CD44^−^ CD25^+^ stage. Rag null cells cannot rearrange TCR genes and lack pre-TCR. Therefore, they fail to form DP T cells and arrest at CD44^−^ CD25^+^ (DN3 stage). When Rag null are transduced with Notch ligand (ICN1), they do not generate DP. If, in addition, they are transduced with a TCRβ transgene, they form DPs and rapidly expand, indicating that pre-TCR signals complement Notch to develop DPs. ICN1 (intracellular Notch 1)-induced bone marrow (BM) DPs cells from both WT and Rag null x TCRβ rapidly expand. In contrast, ICN1-induced Rag null did not, suggesting that pre-TCR signals are required for the proliferation burst that accompanies thymocyte differentiation. Mice repopulated with ICN1-transduced WT hematopoietic stem cells (HSCs) generated extrathymic DP T cells within 3 weeks. In contrast, mice repopulated with ICN1-transduced Rag-2^−/−^ did not generate DP T cells. These findings are consistent with a model in which Notch commits lymphoid precursors to the T lineage; however, pre-TCR signaling is required for the proliferative burst that accompanies thymocyte differentiation.

Recipients of ICN1-transduced HSCs from Rag null mice remained alive for >1 year after transfer. In contrast, all mice receiving ICN1-transduced BM cells from Rag null mice expressing a TCRβ transgene developed T-cell leukemia between 9–11 weeks after transfer. Thus, ICN1-mediated transformation of T-cell progenitors required the expression of a TCRβ chain and development of CD4+ CD8+ T cells [116]. Under physiological conditions, thymocyte proliferation ceases shortly after the cells reach the DP stage, and Notch signaling fails to influence thymocyte development following β selection [117]. In these experiments, three situations may be distinguished: the physiological situation when DNA recombination is followed by cellular proliferation and differentiation, which abates shortly after cells reach the DP stage 2/arrested cell proliferation–differentiation in the absence of the necessary signals delivered by the Notch signaling, plus preTCR 3/cell proliferation–differentiation extending beyond the DP stage in the presence of transduced TCR and constitutive Notch signaling. This situation leads to leukemic transformation but is not preceded by V(D)J recombination as Rag has been deleted. Here, the parallelism with GC-derived tumors apparently fails as there is no DSB at the start of the process. However, it is tempting to speculate that some minor form of DNA perturbation may be present since DP cells are formed and there are DNA modifications that must concur with Rag at this crucial differentiation step. For instance, single stretches of DNA may be induced by cofactors that participate along with Rag in the formation of the pre-TCR, such as the surrogate light chain and CD3 chains. This may well be the case since injection of anti-CD3ε mAb into Rag-deficient mice allows progression of DN thymocytes to the DP stage [118]. For instance, the recombinase cofactor RUNX1 may set a similar course of events in accordance with its frequent involvement in early myeloid and lymphoid leukemias, which has been attributed to its function as a recombinase cofactor and critical regulator of the earliest human TCRδ rearrangement that occurs at a very immature early T-cell precursor (ETP) stage [119]. In summary, the parallelism between the process of leukemic transformation in the GC and in the DN3-DP stage of thymocyte development is apparent and would involve, in both cases, the induction of a DNA damage response (DDR) or elements of it. The checkpoint phase of DDR may be hampered by concomitant cell proliferation or deficiency of factors involved in the DDR, whereas in the absence of cell proliferation, as occurs in the combined absence of Notch and pre-TCR signaling, a checkpoint defect would be inconsequential. Any stimulus enhancing cell proliferation must boost the harm caused by checkpoint defects, thus generating further alterations both in genomic and telomere DNA, leading to tumor development. On the other hand, sustained activation of ICN1, which, in contrast to its abatement under physiological conditions, may persist after cells reach the DP stage, may favor transformation.

## 5. Telomere Complex Alterations Can Directly Induce Cancer

### 5.1. Cancer Induced by the Sheltering Protein TRF2

Overexpression of TRF2 under the control of Keratin5 promoter leads to increased UV-induced tumors as well as spontaneous cancer [120,121]. TRF2 overexpression results in telomere degradation even in the presence of telomerase, but telomere shortening can be rescued by deletion of XPF nuclease. As this nuclease, which is involved in the repair of UV-induced lesions, can also degrade the single-stranded G-overhang at telomeres, it was hypothesized that TRF2 would sequester XPF at telomeres, resulting in deficient XPF elsewhere at the genome, thereby diminishing protection against UV irradiation and UV-induced tumors. However, TRF2 is abundantly expressed in different cancers apart from skin carcinomas, such as breast, liver, and lung carcinomas. On the other hand, TRF2 in the K5-TRF2 model resulted in both UV-induced and spontaneous tumors. In a new approach, these authors made use of successive generations of Terc^−/−^ mice that were crossed with K5TRF2 mice to study the presumed protective role of short telomeres on tumor development. Surprisingly, telomerase deficiency in the K5TRF2/Terc^−/−^ mouse model led to an increased frequency of chromosome aberrations consistent with increase telomere shortening caused by TRF2, which adds to the effect of Terc deletion. However, carcinogenesis was also significantly promoted [120,121]. The findings showed that despite the role of TRF2 in protecting t-loops through ATM inhibition, there was normal activation of the ATM signaling pathway in response to IR, as attested by Chk2 phosphorylation and increased p53 level. It was uncertain whether deficient p53-induced apoptosis might have favored tumor development, as p53 levels were found to be decreased in tumor tissue in two cases as compared to noninvolved surrounding tissue and showed variable levels in other two tumors. Interestingly reversal of telomere shortening was evident in late generations of K5TRF2/Terc^−/−^ mice together with increased intensity of telomere fluorescence, telomere elongation, and other parameters indicative of an ALT mechanism, such as elevated numbers of telomere sister chromatide exchanges (T-SCE) and colocalization of PML with telomeres [122].

The explanation offered by the authors for the promotion of tumor development In these independent models invoked the ability of telomerase in the K5TRF2 telomerase proficient model to provide for continuous cell division, whereas a “DNA damage response (DDR) induced by short telomeres” would be responsible for tumorigenesis in the telomerase-deficient K5TRF2/Terc^−/−^ mice. In both cases, they pointed out that increased chromosomal instability may result from the combination of telomere shortening and a defective NER pathway. In my view, deficient apoptosis through a deficient checkpoint arm of the DDR, as could be the case in the tumors displaying decreased p53 levels, may have contributed to malignant transformation. This was suggested to have resulted from the cancellation of ATM by TRF2. My own suggestion in the case of telomerase-proficient mice is that telomere shortening induced by TRF2 may have led to immortalization through reactivation of telomerase, by a process similar to immortalization after crisis, whereas immortalization in the telomerase-deficient mice should have arisen through activation of an ALT mechanism, as suggested by the intense telomere fluorescence signals, associated PML bodies, and telomere elongation observed in tumoral tissue of G2K5TRF2/Terc^−/−^ not present to the same extent in nontumor surrounding tissue.

Although the role of TRF2 is far from being understood, current knowledge is compatible with this interpretation. Confirming data gathered in the above reports, it has been found that overexpression of TRF2 may cause replication stalling at telomeres and, hence, telomere attrition. On the other hand, induction of ALT by TRF2 has been reported by other authors. SUMOylation of TRF2 has been found to occur at dysfunctional telomeres, inducing elongation via ALT [122]. TRF2 appears to have divergent function in telomeres and extratelomeric DNA. It protects telomeric t-loops by inhibiting ATM and it has been detected at DSB sites next to other DDR factors. A complex interplay between extratelomeric DNA and TRF2 has been described previously. Genomic damage can transiently increase TRF2 expression, whereas telomere damage may lead to increased TRF2degradation and accelerate telomere shortening. TRF2 expression has been found to be elevated in breast and colorectal cancers, whereas loss of TRF2 has been observed in Hodgkin’s disease [122].

### 5.2. The PI3K Pathway and DDR

The PI3K/AKT pathway is connected to the DNA damage response and can be activated following DNA damage. Bozulic et al. [123] reported that Akt/Pkb is activated by one of the central kinases of the DNA damage response pathway, DNA-PK, following double-strand breaks. It has also been shown that pTen, the phosphatase that antagonizes AKT activation, is a target of ATM, the main central sensor of the DDR pathway [124]. The PI3K/AKT pathway is the most frequently mutated pathway in cancer. Some of its individual components, such as p110α and Pten, are among the most frequently mutated genes in cancer; however, many other lesions that activate this pathway are also involved in tumorigenesis (oncogenic Ras, AKT, loss of LKB1, etc.) [125].

The pathway is initiated by the conversion of phosphatidylinositol (3,4) bi-phosphate (PIP2) into the second messenger (PIP3). PIP3 binds PKB/AKT, provoking a conformation change which allows protein PDK1 to phosphorylate AKT at position T308. Full activation of AKT requires phosphorylation of AKT at Ser473 carried out by mTORC2. The phosphatase and tensin analog (Pten) negatively regulates AKT through its phosphatase activity, but Pten mutations outside the phosphatase domain demonstrate an additional role for Pten in the control of chromosome stability. Nuclear Pten binds centromeric protein C (CENP-C) and targets different proteins such as focal adhesion kinase (FAK). Pten can participate in DSB repair through induction of RAD51 and may regulate the cell cycle through the PI3K-p27-CDK2 axis [126,127]. In agreement with the central and many-branched role played by Pten, deletion of this gene represents one of the more effective and faster means of cancer induction. Given the complexity of signaling pathways arising from this central node, numerous studies have been devoted to dissecting the impact of Pten deletion, Akt/Pkb activation, and their downstream targets in tumor development. Phosphorylation of Akt is followed by phosphorylation of GSK3β, FOXO1, and mTORC1.

Some initial studies suggested that transformation in the PI3K pathway was mediated exclusively via mTORC1 activation [128]. Partial reduction of Akt through Akt1 deletion significantly reduced skin carcinogenesis as well as Harvey Ras-induced tumors. Deletion of both Akt1 and Akt2 resulted in almost complete resistance to oncogenic transformation. In subsequent experiments, the same authors observed that Tsc2 deletion led to high mTORC1 activity and enhanced cellular replication, as measured in vitro, while Akt phosphorylation was diminished as a result of a feedback inhibitory loop. On the contrary, increased Tsc2 expression led to downregulated mTORC1 activity, decreased number of foci, and increased Akt activity. From these results, they concluded that Akt-mediated transformation relied mainly or exclusively on mTORC1 activity. However, this conclusion was based on the presumed absolute equivalence of in vitro proliferation foci with oncogenesis in vivo. The latter was not measured in these experiments. mTORC1 controls coordination of ribosome biogenesis and cell growth through its downstream targets S6 and 4E-BP1s. mTORC1 regulation of ribosome biogenesis requires S6 kinase and is mediated by upstream binding factor (UBF), a cofactor of RNA polI [129]. Suppression of UBF in the face of unaltered levels of other proliferation-associated proteins has been shown to lead to apoptosis [130], presumably due to cell proliferation unsupported by lack of protein synthesis. In addition, the inhibition of mTORC1 function by conditional deletion of Raptor, a component of the mTORC1 complex, revealed that phosphorylation of p70S6 and 4E-BP1 was dependent on the cell developmental stage. Consequently, a faulty mTORC1 function is more disruptive for transit, amplifying cells that are actively replicating and need a higher protein supply than stem and quiescent cells. This is reflected in the response of different populations of acute leukemia cells (AML) to mTORC1deprivation. mTORC1 loss leads to apoptosis of mature cells and reduction of tumor burden but does not affect leukemia stem cells (LSCs).

After Pten deletion, mice develop hematological malignancies, the most prevalent of which is T-cell acute leukemia/lymphoma. The incidence of malignancies was decreased in Pten/Raptor double knockouts but incidence was still significant. In contrast, this study did not find acute leukemia/lymphoma development in Pten, Rictor double-deleted mice. Cheng et al. [131] induced mTORC1 activation by conditional Tsc1 deletion. mTORC1 activation induces HSC proliferation followed by exhaustion. It was shown that increased ROS levels were responsible for HSC exhaustion because treating the mice with the antioxidant N-acetylcysteine prevented bone marrow hypocellularity and restored reconstitution capacity. However, in contrast to Pten deletion, which, like Tsc1 deletion, results in mTORC1 activation, leukemogenesis did not follow HSC exhaustion in Tsc1 KO HSCs. Contrary to the restoration of repopulating ability by N-acetylcysteine observed in Tsc1 null-HSCs, the loss of repopulation ability caused by Pten deletion could not be restored by antioxidant treatment [132]. These discrepancies might be due to the different experimental models used, but are more likely due to a greater impairment of HSC function of Pten null HSCs.

Similarly, in leukemias associated with Pten chromosomal translocations, it has been repeatedly observed that inhibition of mTORC1 signaling by rapamycin delays the onset of leukemia and reduces tumor burden but it does not affect the leukemia stem cell. From a clinical perspective, it is worth noting that mutations in TSC1 or TSC2 are responsible for tuberous sclerosis, which is associated with hamartomas rather than carcinomas. Nevertheless, mTORC1 contributes to malignant transformation induced by Akt activation because rapamycin decreases the incidence of T-cell lymphomas in murine models using constitutively active AKT [133]. Thus, the high efficiency of the PI3K/AKT/Pten pathway in promoting leukemia may result from the convergence of oncogenic signals mediated by several downstream targets of this pathway. This is consistent with the concept that cell proliferation may act in concert with deregulation of the mitotic cycle to elicit oncogenic transformation.

GSK3β is a downstream node of the PI3K pathway from which divergent signaling for HSC self-renewal and lineage commitment emanate. Activated Akt phosphorylates GSK3β, leading to its inhibition and subsequent activation of Wnt-βcatenin signaling which enhances HSC self-renewal. Concomitantly, GSKβ inhibition also activates mTORC1, promoting lineage commitment. By simultaneously stimulating the Wnt-βcatenin pathway with inhibitors of GSKβ CHIR99021 or lithium and inhibiting the mTOR pathway with rapamycin, Huang et al. [134] were able to maintain long-term multilineage hematopoiesis in cytokine free cultures treated with both inhibitors. These culture conditions allowed tri-lineage hematopoietic reconstitution and gave rise to mature myeloid cells, contrary to control cells cultured in a standard cytokine cocktail. A higher percentage of c-Kit+ quiescent cells in G0 as well as a small increase in S/G2/M, indicating some increase in cell growth, were seen in the treated cultures. Transplantation to secondary recipients confirmed the improved preservation of HSC function as assessed by a higher chimerism detected in recipients of cells treated with inhibitors [134]. Although HSC proliferation and subsequent exhaustion mimic similar outcomes induced by Pten deletion, there are important differences that have been revealed by these experiments. First, HSC exhaustion induced by GSKβ inhibition is reached more slowly and becomes evident only through serial transplants and competitive repopulation assessment. More importantly, Pten loss or constitutive Akt activation leads to the rapid development of leukemia which has not been observed so far in GSK3β-rnai transplanted mice or lithium, whose use in the treatment of bipolar disorders has never been associated with increasing risk of malignancies [135]. In addition, the preservation of physiological tri-lineage hematopoietic differentiation induced by Wnt-β-catenin signaling contrasts with the biased expansion of immature myeloid progenitors subsequent to HSC mobilization observed after Pten deletion or Akt constitutive activation [133]. Again, exit from the stem cell pool appears as a step to malignant transformation. These observations suggest that cell proliferation induced by mTORC1 is not sufficient to induce transformation, and point to an essential oncogenic contribution from FOXO, another downstream target of activated AKT.

The FOXO group of transcription factors (FOXO1 (FXHR), FOXO3a (FKHRL1), and FOXO4 (AFX)) act downstream of the PI3K/AKT pathway [136] Interestingly, sites of Akt phosphorylation at threonine 308 (pAKT^Thr308^) and Serine 473 (pAKT^Ser473^) are essential for full Akt activation, but pAKT^Ser473^ is dispensable for AKT-mediated phosphorylation of TSC2 and GSK-3β and is required for phosphorylation and inactivation of FOXOs [137].

FOXO proteins are normally present in an active state in the cell nucleus where they are involved in cell cycle arrest or apoptosis (depending on the cell context), cell cycle progression through induction of cdk inhibitors p27/Kip1 and p57 as well as long-term survival and control of factors regulating stemness such as OCT4 and SOX2 [138]. Nuclear exclusion (inactivation) of Foxo3a is associated with the first step in hematopoietic differentiation, as Foxo3a is present in the nucleus of freshly isolated Foxo3a^+/+^ CD34^−^ KSL cells (HSCs) but appears in the cytoplasm of freshly isolated Foxo3a^+/+^CD34^+^ KSL cells (progenitors) [138]. Clonogenic assays show that Foxo3a deficiency impairs the long-term (16 weeks) reconstitution ability of CD34^−^ KSL (HSCs) but not the short-term reconstitution capacity of CD34^+^ KSL cells (progenitors). The decline of repopulation efficiency of HSCs is associated with loss of quiescence and decreased levels of cell cycle inhibitors p27 and p57 in Foxo3a^−/−^ CD34^−^ KSL cells [138]. Active FOXOs play an essential role in the maintenance of stemness; consequently, loss of stemness and exit from quiescence can result from Foxo nuclear exclusion and inactivation.

**Figure 1:**
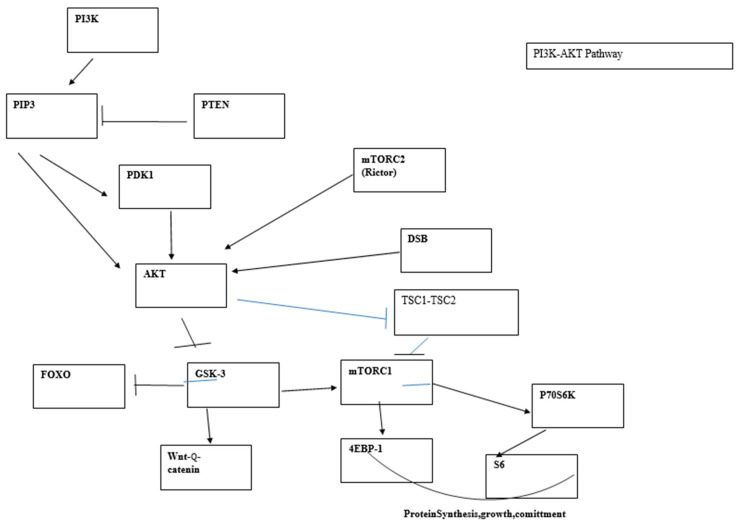
Main circuits of the PI3K/AKT pathway and its connection to DSBs.

Finlay et al. [139] showed that “PDK1 has an obligatory function in controlling the phosphorylation and transcriptional inactivation of Foxo1, 3a and 4 in Pten-null cell” and that “Pten null T cell progenitors cannot transform or develop into invasive and fatal T lymphoma without PDK1”. Other findings lend further weight to the role of this oncogenic mechanism, such as the requirement of the mTOR complex 2 for development of prostate cancer in Pten null mice [140] or the suppression of leukemogenesis in Pten null mice by concomitant deletion of Rictor (an essential component of mTORC 2) [141] (there has been a hot debate on the role of PDK1 in Akt phosphorylation at Ser473). Other kinases referred to as PDK2 activity, and more recently mTORC2, have shown to be responsible for AktSer473 phosphorylation [142]. Nevertheless, this does not invalidate the conclusions of Finlay or the ideas defended here that boil down to the paramount role of Foxo inactivation induced by pAktSer473 on T-cell lymphomagenesis [141]. Magee et al. showed that Rictor deletion with the subsequent suppression of mTORC2 prevented leukemogenesis and HSC depletion in Pten-deleted adult mice [141]. The expression of myr-AKT in HSCs that is associated with HSC mobilization and exit from the stem cell compartment must correlate with inactivation and cytoplasmic translocation of Foxo 3a (the main form of Foxo in hematopoietic cells) as it has been observed that transition of HSCs to progenitors is associated with inactivation and cytoplasmic translocation of Foxo [138]. In normal HSCs, the activity of Akt is attenuated as required for quiescence, whereas Akt activation rises in normal granulocyte-macrophage progenitors (GMPs). Paradoxically, Sykes et al. observed growth suppression in the AML-AF9 leukemia model [143] after enforced activation of AKT in HSCs by means of the MSCV-IRES-GFP-myr-Akt construct. The leukemia stem cell (LSC) shared the immunophenotype of GMPs (lineage ^low^, c-Kit ^high^, FcγRII/III+, CD34+) but had the attenuated Akt pattern of a normal stem cell associated with active nuclear Foxo. Since this expression pattern seems to be related to maintenance of stemness, it is probably a general feature of the transformation process rather than a specific feature of the AML-AF9 leukemia. This suggests that the differentiation of HSCs into committed progenitors is an obligatory step in the process of transformation after which the committed progenitor must undergo a partial reversion in order to acquire the attenuated pattern of Akt expression that supports quiescence. A possibility was that activation of mTORC1 induced by pAKT with subsequent differentiation of tumor cells was responsible for this paradoxical beneficial role of pAKT in leukemic growth. Akt activates mTORC1 by relieving inhibition of mTORC1 by TSC2. As this explanation was ruled out under rapamycin treatment, other actions of pAkt, independent of mTORC1 activation, must be responsible for myeloid maturation and growth inhibition. Given the substrate selectivity of pAKT^Ser473^, the involvement of Foxo inactivation was an obvious choice. This was confirmed by inhibiting Foxo3a with shRNA in MLL-AF9 as well as in AML cell lines that do not carry MLL translocations [143]. Foxo inhibition lowered tumor growth and induced myeloid-maturation-related death, but Foxo inhibition does not affect the generation or survival of leukemic stem cells. Thus, pAKT^Ser473^ induced Foxo3a cytoplasmic translocation and exit from the HSC compartment (commitment) concomitantly, but generation of the leukemia stem cell involves reversion of a committed cell to the attenuated AKT pattern of the normal stem cell. In other words, HSC differentiation and subsequent leukemic transformation are two outcomes of Foxo3a signaling that affect tumor biology in opposing ways, but once leukemic transformation is established only Foxo’s deactivation role on cell differentiation is observed. Obviously, deletion of Foxo in AML-F9 transplanted Cre^+^ recipient mice extended latency and survival due to differentiation-related cell death, but the majority of mice eventually succumbed to leukemia, suggesting that, independently of its effect on myeloid maturation and apoptosis, Foxo3 depletion cannot eradicate LSCs after transformation. Collectively, these results imply that putative transformation of a stem cell requires a previous step of differentiation (Figure 1).

Hu et al. [144] revealed an Akt-independent mechanism of Foxo3a inactivation dependent on IκB kinase (IKK). Constitutive expression of IκB kinase leads to Foxo3a inactivation and nuclear exclusion, cell proliferation, and tumorigenesis, a clear indication that the proleukemogenic role of Foxo3a predominates over its antileukemic effect. Degradation of IκB_α_ by IκB kinase is accompanied by the activation and nuclear translocation of NF-κB, which is known to be associated with the upregulation of cyclin D1 and cell proliferation. However, tumorigenesis was attributed to Foxo inactivation because cell clones transfected with, and expressing, IκB kinase induced mammary tumors in nude mice which could be suppressed by re-expression of Foxo3a [144]. The interplay between Foxo and NF-κB may be crucial for the integrity of the cancer stem cell and this interplay may be influenced by telomere alterations through the demonstrated ability of telomere protein Rap1 to bind to IKK, thereby promoting degradation of IκB and subsequent translocation of NF-κB to the nucleus [145]. The same outcome (IKK activation and NF-κB nuclear translocation) can be elicited by DNA double-strand breaks (DSBs) but not by proinflammatory stimuli [146]. Following DSBs, ATM can activate IKK, leading to the degradation of IκB and release of bound NF-κB which translocates to the nucleus. In contrast to upregulation of cyclin D1 expression, antiapoptosis, and cell proliferation associated with nuclear NF-κB, nuclear FOXO drives cell cycle arrest or apoptosis, whereas cytoplasmic Foxo drives cell proliferation. Under physiological conditions, AKT activity is low in quiescent cells and it is associated with retention of FOXO factors in the nucleus, where they upregulate expression of target genes that control cell cycle, i.e., p27kip1, Rb2(p130), mitosis (cyclin B and polo-like kinase, metabolism, or apoptosis (Fas ligand and Bim)) [137,138]. Some of these phenotypic traits, like upregulation of p27 and cell cycle arrest, can also be induced by re-expression of Pten. In contrast, Pten deletion and subsequent Akt activation led to FOXO inactivation and nuclear exclusion, and presumably cycle control deregulation. The proapoptotic function of Foxo proteins is in great part dependent on their collaboration with E2F1 protein. This collaboration is required for E2F1-induced apoptosis but not for E2F1-induced proliferation. A protective DNA damage reaction increases apoptosis through E2F1-Foxo because it stabilizes E2F1 through ATM and Chk2 phosphorylation, whereas the Foxo-dependent arm of E2F1 signaling in charge of apoptosis was found to be reduced in most tumor types examined relative to counterpart normal samples. These findings highlight the relevance of the cross-talk between the oncogene-induced DNA damage reaction and the PI3K pathway [147]. Pin1, a target of E2F1 essential for Neu/Ras-induced transformation of mammary epithelial cells through activation of cyclin D1 [148] (to be discussed later), has also been reported to induce nuclear accumulation of NF-κB.

The simultaneous cytoplasmic translocation–inactivation of Foxo leading to deficient apoptosis, differentiation (exit from the stem cell pool) and prosurvival effect, and induction of cell proliferation by nuclear translocation of NF-κB appear to reinforce each other in the transformation and survival of the cancer stem cell.

In summary, downstream targets of the PI3K pathway that impinge on cell cycle control and skewed differentiation appear to be more relevant in oncogenesis than those that stimulate cell proliferation, although cell proliferation may still be required to elicit transformation. Interconnection between DDR and PI3K signaling occurs at different levels and may be crucial for transformation, as discussed in the next section.

### 5.3. T-Cell Lymphoma Development Following PI3K/AKT Signaling Is Triggered by V(D)J Recombination Which Coincides with the Tumor Cell Differentiation Stage and Resistance of Neonatal Cells to Leukemia Development

The Ser473 AKT mTORC2 site required to inactivate Foxo3a is also required to mobilize HSCs, as demonstrated by the decrease in the S/G2/M fraction of HSCs after Pten deletion when Rictor, a component of mTORC2, is deleted. The mobilization of HSCs has an important proleukemogenic effect, as demonstrated by the antileukemic role of Rictor deletion in Pten-deficient cells [141]. Rictor deletion, but not rapamycin treatment, reduced HSC mobilization after Pten deletion. Rapamicin treatment and Rictor deletion both reduced HSC proliferation after Pten deletion and additively reduced the severity of myeloproliferative disorder following Pten deletion.

An experiment by Xue et al. [149] revealed a special form of cancer resistance linked to cell or organ immaturity, which may underlie the resistance of stem cells to cancer development. They described a different behavior of HSC in neonatal mice compared to that in adult mice in their response to Pten deletion. When Pten deletion was performed 2 days after birth, the expected increase in HSCs number was only observed in 8-week-old mice (>40-fold) but it had no effect on the number of HSCs in 2-week-old mice. Overall proliferation of unfractionated bone marrow cells at 2, 3, 4, and 5 weeks of age was not affected by Pten deletion. On the other hand, control neonatal HSCs divided more actively than adult HSCs, with transition from neonatal to adult phenotype taking place between 4–5 weeks. In transplantation experiments, Pten-deficient neonatal (before 6 weeks of age) HSCs showed long-term multilineage reconstitution lasting from 16 to 24 weeks, and even this constraint (16–24 weeks) appears to reflect their maturation to an adult phenotype. In contrast, Pten-deficient adult HSCs showed only short-term reconstitution lasting less than 16 weeks. It was also found that Pten prevented leukemia of adult but not neonatal mice. The fact that adult mice succumbed to leukemia 12 weeks after Pten deletion made it difficult to test whether neonatal mice were resistant to leukemia, since their HSCs would mature before the onset of leukemia. To circumvent this difficulty, pIpC was administered to Mx1-Cre, Pten^fl/fl^, p53^−/−^ mice, and littermate controls lacking Mx-1-Cre at 2 days or 6 weeks after birth. All mice in the 6 weeks group died before 20 days of pI-pC treatment, whereas at 21 days after pI-pC treatment, none of the doubly deficient Pten, p53 neonatal mice showed any sign of illness and did not die until 44 to 60 days after pI-pC treatment. Additionally, comparison of PI3K kinase activation in Pten-deleted neonatal and adult mice showed that Pten deletion increased phosphorylation of most substrates of the pathway (AKT, S6, and GSK3β, but not MAPK or AMPK) in LSK from adults but not neonatal mice. Nevertheless, HSCs from neonatal mice can activate this pathway, as demonstrated in in vitro cultures with 2% bovine serum. AKT phosphorylation at Ser473 distinguished Pten-deficient HSCs from HSCs that are dividing under physiological conditions, and Rictor deletion, which suppresses mTORC2 function, substantially reduced AKTSer473 phosphorylation, consistent with mTORC2 being less active in neonatal HSCs. Rapamicin did not affect AKTSer473 but Rictor deletion modestly reduced mTORC1 (modest reduction in S6 phosphorylation). Proliferation of HSCs after Pten deletion depends on mTORC1 as it is reduced by rapamycin. In contrast, Rictor deletion in Pten null HSCs reduced the frequency of HSCs in cycle. Also, in adult mice, Rictor deletion significantly reduced MPPs but not other lineages. All these findings show that mTORC1 induces HSC proliferation–differentiation and is the main regulator of these activities under physiological conditions, whereas Pten deletion induces HSC proliferation and mobilization of HSCs through mTORC2-dependent and -independent mechanisms that do not mimic physiological HSC self-renewal. These findings are consistent with the role previously described of AktSer473 in relation with Foxo. Moreover, Rictor deficiency in Pten null cells restored long-term reconstitution ability (of myeloid and T cells, although not B lymphoid cells), which suggests that loss of long-term reconstitution is associated with HSC mobilization (and accelerated differentiation to MPPs since Rictor deletion reduced MPP number) and, in turn, prevention of leukemia development by Rictor deletion correlates with restored repopulation efficiency due to decreased HSC mobilization and early differentiation to MPPs.

Therefore, the resistance of neonatal cells to leukemia development appears to depend on a deficient mTORC2 activity in neonatal HSCs. However, the question remains whether antileukemic protection afforded by deficient mTORC2 activity may do so by preventing exit from the stem cell pool and loss of self-renewal potential associated with stem cells.

In addition, the work of Xue et al. [149] demonstrates the role of a DNA damage response in the initiation of oncogenesis through the PI3K/AKT pathway. T-cell thymomas that develop in Pten-deficient mice are CD4^+^ CD8^−^ mature T-cell thymomas. These researchers observed that premalignancy only starts at 9 weeks of age in the preceding stage of double-positive (DP) thymocytes and is accompanied by classical markers of a DNA damage reaction such as phosphorylation of DDR substrates p53, chk1, and chk2 in DP thymocytes. These markers were highly phosphorylated in w.t. thymocytes consistent with the stage where V(D)J recombination occurs (it was negligible in CD4SP and naïve w.t. T cells). Pten-deficient DP thymocytes expressed higher levels of these proteins but not of γH2AX (no thymocyte populations expressed γH2AX). They also expressed higher levels of p19 and p21. AKT phosphorylation was not detected before 6 weeks of age in Pten-deficient T cells. Concomitantly, Foxo3a was heavily phosphorylated in DP T cells but not in SP or naïve T cells of Pten-deficient 9-week-old mice. GSK3β was only slightly activated but S6K was unaffected. They concluded that, although Pten is lost since birth, premalignancy does not start until mice are 6 weeks old and activation of AKT in DP cells differentially affect downstream targets of the PI3K pathway.

DP thymocytes from both controls and Pten-deficient cells were Ki67 positive (a marker of non-G0 cells) but did not proliferate. The most striking changes were seen in the expression of p27 that was expressed at high level in w.t. DP thymocytes and was significantly reduced in Pten-deficient DP thymocytes [149].

The association of stem cell resistance to leukemogenesis with the need for a short period of organism maturation before a cell becomes vulnerable to cancer transformation points to obscure mechanism/s of anticancer protection linked to the cell immaturity, which may underlie the observation that, contrary to a widespread assumption, there are few true stem cell cancers. On the other hand, the synchronous appearance of premalignancy at the DP stage suggests that the DNA strand breaks that occur at this developmental stage along with V(D)J recombination initiate a DDR that, in conjunction with Pten deficiency, is responsible for malignant transformation. The role of concomitant DNA damage reaction with defective cell cycle control is consistent with the finding that Pten^−/−^ cells display a defective checkpoint response in response to ionizing radiation (imputed to the ability of AKT to phosphorylate CHK1 since no phosphorylated mutant could complement the checkpoint defect) [150].

Different circuits of the PI3K pathway appear to support oncogenesis in a double direction: 1. By promoting escape from the stem cell compartment and interference with telomere protective mechanisms of stem cells; 2. Through production of factors such as NF-kB that may help survival of cancer stem cells. Interestingly, the latter function may be induced through telomeric protein Rap1.

## 6. Correlation with Clinical Data

A study using whole genome sequence analysis of 2658 genomes [151,152] and their matching normal tissues across 38 tumor types (of these, 2583 were considered high-quality samples) demonstrated that driver mutations in the TERT promoter were by far the most frequent of noncoding mutations as well as the most frequent across different tumor types (14 cohorts out of 38) [152]. Overexpression of the telomerase gene either through point mutations in the promoter, hitching TERT to active regulatory elements such as viral enhancers, or through gene amplification was detected in 270 samples. In addition, loss-of-function mutations in the ATRX and DAXX genes that result in telomere elongation through ALT were detected in 128 tumors, of which 71 had protein-truncating mutations. By analyzing 12 features of the telomere sequence, this study suggests that there may be other unknown mechanisms, in addition to telomerase re-expression and ALT, for telomere maintenance and, therefore, extension of cell survival. Such a mechanism, independent of both telomerase and ALT, can explain the occasional finding of immortal cell lines maintained in the absence of telomerase expression and ALT-associated promyelocytic leukemia bodies [153].

Only 13% (785 out of 5913) of driver point mutations in pan-cancer-analysis of whole genomes (PCAWG) consortium were noncoding. However, 25% of PCAWG tumors had at least one putative noncoding driver mutation, and one-third (237 of 785) were in the TERT promoter. This figure may underestimate the real frequency as it was thought that only around 50% of PCAWG tumors had sufficient coverage to call a mutation at the two TERT promoters (among the cohort with no known drivers, further analysis identified TERT mutations in six hepatocellular carcinomas and two biliary cholangiocarcinomas). Aneuploidy and chromosome instability were frequently observed in this cohort.

TP53 mutations were found in 954 tumors; 77% of them had biallelic mutation and 17% of patients had mutations in cancer predisposition genes and DNA-damage response genes such as BRCA. Oncogenes including KRAS, BRAF, and PIK3CA were also mutated. Some frantic episodes of genomic instability that arise as a single event are frequent oncogenic drivers: chromoplexy, which entails the occurrence of simultaneous double-stranded breaks in different chromosomes, was identified in 17% of samples. It may give rise to reciprocal translocations. In 48 thyroid adenocarcinomas, 13 fusion (31%) genes were attributed to chromoplexy and another 31% contained chromoplexy footprints. Kataegis, a focal hypermutation process associated with clustered nucleotide substitutions biased towards a single DNA strand, leads to deletions and complex rearrangements. Chromothripsis occurred in 587 samples (22.3%). It was frequently associated with TP 53 driver mutation. Most of the coincident driver events were amplifications events. In liposarcoma, amplification of TERT was observed in 4 out of 19 cases. In chromophobe renal cell carcinoma, breakpoint adjacent to TERT led to an 80-fold increase in TERT expression.

Driver structural variants may act by inactivating a tumor suppressor or deregulating a gene through fusion or juxtaposition. The authors distinguished significant recurrent breakpoints (SRBs) from significant recurrent juxtapositions (SRJs) based on the variability of breakpoints on the other side. Eight were tightly clustered and involved known oncogenic fusions. The remaining 45 were not clustered. It was found that late replication timing predicted fragility-associated SRBs rather than existing annotations, thus identifying 12 fragile-like SRBs. The remaining 33 single copy number alteration comprised 14 amplifications, 8 deletions, and 11 copy number neutral events. Five of the eight deletion-associated SRBs were associated with biallelic inactivation of nearby tumor suppressors, compared to none of the 12 fragile-like SRBs [152]. These findings highlight the overwhelming potential oncogenic role of telomere alterations, which clearly surpass those arising from regions of the genome that are either late replicating or containing repetitive elements. Furthermore, activation of telomere maintenance may be responsible for a substantial fraction of other cancers, such as those derived from oncogenes and diverse forms of replication stress. In addition, telomere oncogenic pathways may arise through nonmutational mechanisms such as telomere position effects or novel regulators. Re-expression of telomerase as a final step of telomere attrition in crisis or telomere maintenance through ALT may be just one of several mechanisms. Finally, downstream targets of telomere oncogenic pathways such as TGFβ may also be directly responsible for some cancers.

## 7. Further Comments and Conclusions

The preceding discussion is aimed at supporting the proposal that oncogenes and other cancer-inducing events, including mitotic disruption and chromosome instability, may lead to disturbances in the telomere complex, which are ultimately responsible for oncogenic growth signaling through known or still-unknown mechanisms of telomere maintenance. A faulty repaired DNA may lead to mutated DNA, leading to enhanced cell proliferation or, if unrepaired, to cell death, but it would be the subsequent checkpoint defect perhaps boosted by stimulation of cell proliferation that would directly induce cell cycle deregulation and telomere dysfunction ultimately responsible for malignant transformation. Furthermore, even in the absence of direct disturbance of the cell cycle, DNA alterations may directly impact the telomere. For instance, it has been reported that deficiency of DNA mismatch repair increases the rate of telomere shortening [58].

The above observations regarding the proleukemogenic role of loss of quiescence, the frequent initiation of cancer following a DNA damage reaction in a committed cell, and the rare description of true stem cell tumors suggest that stem cells are much less susceptible to DNA damage reactions that are followed by malignant transformation. However, from the earlier stages of lineage commitment, the cell becomes vulnerable to malignant transformation although cancer transformation may require the acquisition of stemness by a committed cell. One important characteristic that separates stem cells from the earliest committed cells is the capacity for indefinite self-renewal, which appears to be linked to a mechanism of telomere maintenance that is, probably, stem-cell-specific. This privileged situation of the stem cell could be lost rapidly through stem cell mobilization. Moreover, stem cell mobilization might be caused by a mechanism that interferes with normal telomere maintenance of the stem cell, for instance, Myc activation of TERT. On the other hand, malignant transformation implies the sudden acquisition of a mechanism for indefinite telomere maintenance that the committed cell did not possess previously. Many studies indicate that the generation of such mechanism requires sustained erosion or other form/s of telomere damage, which may be elicited through different types of DDR. Evidently, DDR occurs more frequently during tissue turnover and differentiation than in the stem cell compartment and may likely lead to disturbances of the telomere complex (erosion, ALT, gain-of-function mutations at the TERT promoter). One of the probable outcomes is telomerase re-expression (or one of its downstream targets), which has been demonstrated to induce transformation independently of its canonical enzymatic activity. All this suggests two interpretations that, far from being mutually exclusive, may be interdependent:

1. Different types of DNA damage reaction might converge on distinct modes of telomere alteration that could induce transformation independently of canonical telomere maintenance but be associated with it (i.e., telomerase can induce transformation independently of telomerase activity, for instance, through its downstream target TGFβ and TGFβ target JunB [154]). Distinct oncogenic pathways are initiated by different telomere lesions caused by diverse forms of DNA damage, which can result in transformation through the emergence of new mechanisms for telomere maintenance. This is consistent with the fact that radical changes in telomere maintenance are associated with cancer transformation. Thus, normal stem cells might be protected from direct cancer transformation by mechanism/s of telomere protection (or protection from DNA damage) that are more efficient in stem cells than in committed cells (telomerase expression or others not well known, such as the capacity for chromosome healing that has been described in mouse embryonic stem cells) [155]. At any rate, telomere alteration occurs much less frequently in stem than in committed cells. However, there are rare cases of telomerase oncogenic pathways starting directly in stem cells (primary defects of the telomere complex). One such case is represented by Beckwith–Wiedemann syndrome, which is associated with an 800-fold increased risk of childhood neoplasms, where multiple tumors arise in different organs or even in the same organ. Overexpression of telomerase reverse transcriptase (TERT), defective TGFβ signaling with epigenetic silencing of β2 spectrin, an SMAD adaptor for TGFβ signaling, are involved in the etiology of this syndrome [7]. Another case in point in the area of molecular cancer modeling were the reports by Passegué et al. [8] and Santaguida et al. [9] that JunB inactivation recapitulates aspects of human malignancies, including chronic myelogenous leukemia. It was described that JunB-deficient HSCs had impaired reconstitution ability compared to control HSCs owing to a lower fraction of quiescent cells. When transplanting equal numbers of CD34^−^/Flk2^−^ LSK cells into irradiated recipients, those from JunB-deficient mice lost reconstitution ability due to their rapid expansion and differentiation into progenitors. After isolating HSCs to near purity using CS150^+^/CD48^−^/Flk2^−^, it was observed that the HSPCs compartment of JunB-deficient mice was expanded by an increase in the CD48^+^/Flk2^−^ population. JunB-deficient CD150^+^/CD48^−^/Flk2^−^ displayed higher repopulation ability than controls, which was apparently caused by an increased production of myeloid cells that ultimately led to the appearance of MPD. Here, it is interesting to point out how rapidly stem cells differentiate into progenitors and therefore lose their indefinite self-renewal ability, and they almost immediately transfer this capacity for indefinite self-renewal to their myeloid progeny. It is intriguing that the expanded tumor population has a granulocyte-macrophage (GM) phenotype [8,9] rather than the preceding common myeloid phenotype, suggesting that transformation does not arise directly at the stem cell stage. Nevertheless, the MPD could only be transplanted to secondary recipients by HSCs; therefore, this was considered true stem cell leukemia even though it had the GMP phenotype and transformation could have occurred in the immediate post-stem cell stage. JunB deficiency was associated with significant reduced expression of Pten, Hoxb4 and Hoxb9, Notch1, and Hes1 and Hes5 in Flk2^−^ LSK cells, but only Hes1, a downstream target of the TGFβ pathway, remained significantly decreased in the LT-HSC population (Hes1 is a downstream target of TGFβ and Notch). When JunB-deficient cells were incubated in OP9 stromal cells expressing the Notch ligand delta-1, Hes1 was not significantly induced. Incubation of JunB-deficient cells with recombinant TGFβ resulted in significant reduced expression of three direct transcriptional targets of the TGFβ pathway (Hes1, Smad7, and the CKI p57). It has been shown that the oncogenic effects of telomerase re-expression can be mediated by reduced TGFβ expression [154]. This indicates that JunB deficiency engages downstream signaling similar to Beckwith–Wiedemann syndrome and telomerase itself. Therefore, these arguments can be extended to many other oncogenic pathways mediated by JunB deficiency or activator protein 1 (AP-1) that engages and/or shares final downstream targets with the telomerase pathway. On the other hand, it would not be surprising that future research unveils common pathways of preneoplastic telomere alteration in these tumors (here referred to as true stem cell tumors) and a subset of childhood/immature tumors.

**Figure 2:**
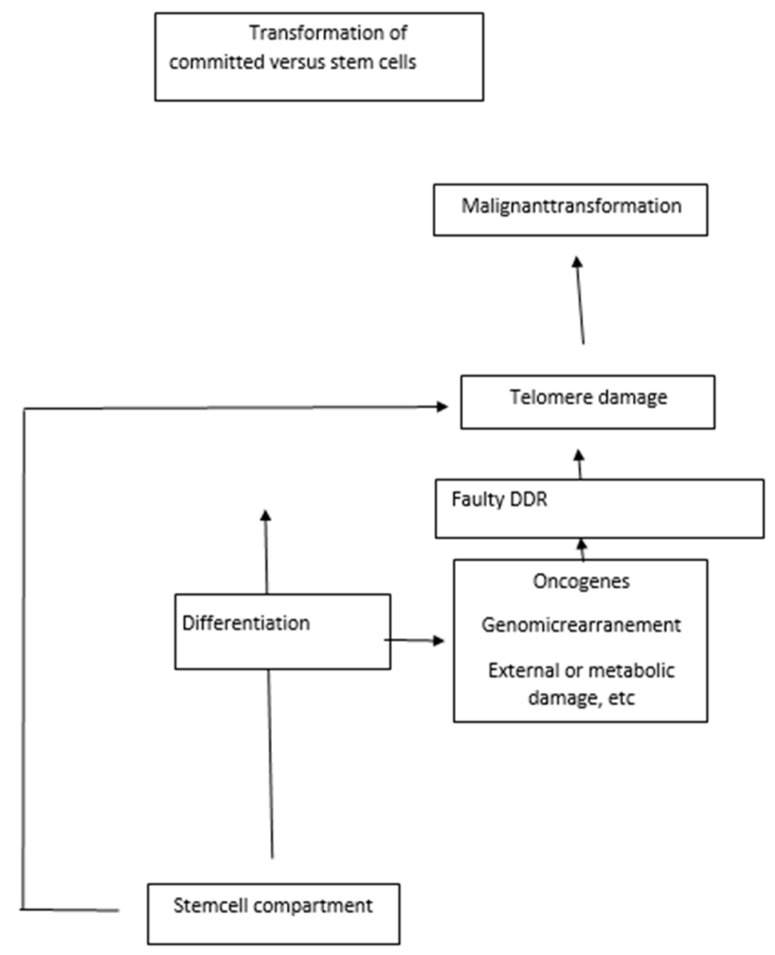
Different routes to malignant transformation are followed by normal stem and committed cells.

According to the above criteria, there are two main oncogenic routes: one initially triggered by DNA damage reactions that converge on telomere alterations ultimately responsible for oncogenic signaling (this mechanism would be preferentially associated with tumors originating in relatively mature cells); and another one that is initiated by mechanism directly related to telomere maintenance represented by tumors of true stem cell origin. However, this may be a simplification because there are oncogenes that may potentially act through both routes. For instance, overexpression of Tcl1 has been shown to cause B-CLL by inhibiting AP-1 (containing JunB) and enhancing NF-κB [156] (Figure 2).

## 8. Future Directions

1. The former observations raise an enigmatic question: How can these pathways that can be initiated by the telomerase pathway (including its downstream targets such as JunB) ultimately give rise to telomerase expression as it is re-expressed by the cancer stem cell? A clue to this question was provided by Chen et al. According to their study in Beckwith–Wiedemann syndrome tumors and the murine TGFβ/Smad3 disruption tumorigenic model, formation of a β2SP/SMAD3/CTCF protein complex is TGFβ-dependent and its disruption in these tumors increases TERT levels. Overexpression of ectopic β2SP and SMAD3 significantly decreased TERTmRNA levels in SNU398 cells and a TGFβ inhibitor blocked this effect, suggesting that β2SP/SMAD3 decreases TERTmRNA levels in a TGFβ-dependent manner and vice versa [7].

2. DNA–telomere interaction is a two-way road. Anomalous DNA damage responses result in telomere damage and the emergence of new telomere maintenance mechanisms. Afterwards, signaling arising from modified telomeres/ALT, independent of telomere maintenance, may contribute to the survival of the cancer stem cell. Although we discussed some examples of the role of telomeric proteins in oncogenic pathways, such as Rap1 induction of NF-κB nuclear translocation, this second type of interaction is largely unexplored.

3. An alternative and non-mutually exclusive explanation for the resistance of stem cells to direct cancer transformation is that it would not be dependent (at least not only) on its more efficient telomere maintenance of telomere integrity but might also be linked to the self-renewal capacity of normal stem cells. Indefinite self-renewal is an exclusive property of both normal stem and cancer stem cells. A clear gradient can be traced in repopulation potential from long-term HSCs (LT-HSCs) to short-term HSCs to multipotent progenitors (MPPs), and diminished repopulating ability has been found to be associated with the propensity for tumorigenesis. A population of HSCs called dormant HSCs (dHSCs), which contains the highest number of quiescent cells within this compartment, has been identified recently. Retinoic acid (RA) metabolism is characteristically enriched in this population compared to that in downstream multipotent progenitors. Peptide GPCR and prostaglandin synthesis were also enriched in dHSCs. These cells transition more slowly than other HSCs toward active HSCs (aHSCs) and show a higher expression of retinoic-acid-induced genes. All-transretinoic acid (ATRA) treatment maintained the dHSCs compartment and increased quiescence. Thus, according to the concept that loss of HSC quiescence facilitates tumorigenesis, treatment of HSCs with ATRA and/or prostaglandin E2 should exhibit tumor suppressor activity [157]. This prediction has already been fulfilled after the discovery of Pin1 inhibition by ATRA [158]. Pin1 enhances cell proliferation by induction of cyclinD1 [159] and regulates the expression of numerous mitosis-specific phosphoproteins [160] whose levels change during the cell cycle in normal cells but not in cancer cells [161]. Moreover, ATRA blocks multiple cancer-driving pathways and suppresses the growth of hepatocellular and breast cancer [160]. These observations provide an explanation for the association of enhanced stem cell quiescence, preservation of repopulation potential, and cancer suppression. It is tempting to speculate that telomere protection mechanisms of stem cells might, indirectly, provide antitumorigenic protection to their progeny. This type of anticancer resistance might underlie the resistance of immature stem cells to transformation induced by PTEN-loss reported by Xue et al. [149].

## Figures and Tables

**Table 1:** Components of the DNA damage response responsible for telomere dysfunction and cancer.

	Telomere Dysfunction	Cancer Predisposition
* P53	Yes [16]	Yes [17]
Rad 9	Yes [18]	Yes [18]
Rad 51	Yes [19,20]	Yes [21]
Rad 52	Yes [22]	Yes [22,23]
Rad 54	Yes [24]	Yes [25,26]
c-Abl	Yes [27,28]	Yes [29]
BRCA1-BRCA2	Yes [30,31]	Yes [32]
** PARP-1	Yes [33,34,35]	Yes [36]
*** DNA-PK_cs_	Yes [28,37]	Yes [38]
Artemis	Yes [28,39]	Yes [40]
Cerunnos	Yes [41]	Yes [41]
Ku70-Ku80	Yes [42,43]	Yes [43,44]
Ligase IV	Yes [45,46]	Yes [46]
Fanconi Anemia	Yes [47]	Yes [46]
^ CtIP	Yes [48]	Yes [48,49]
Rad17	Yes [50]	Yes [51,52]
9-1-1 complex	Yes [53]	Yes [53]
14-3-3 complex	Yes [28]	Yes [54]
CTC complex	Yes [55,56]	Yes [55,56]

Notes regarding Table 1. * (p53) In children, telomere length was found to be shorter in carriers affected with cancer than in unaffected carriers and with respect to controls. The same pattern was observed in adults. Telomere attrition between children and adults was faster in the carriers than in the controls. These results support the role of Mdm2-SNP309 as a genetic modifier of LFS. The finding of accelerated telomere attrition in LFS patients suggests that telomere length could explain the earlier age of onset in successive generations of the same family with identical TP53/MDM2-SNP309genotypes. ** (PARP1) The role of PARP1 in telomeres is controverted but it is acknowledged that it is required for the maintenance of damaged telomeres and associates with R-loops to promote their resolution and genome stability. According to [35], it may not have an effect on telomere length but may affect telomere dysfunction. However, its role in cancer is not well understood. In p53, w.t. PARP overexpression correlated with high expression of stem cell markers in colorectal tumor samples as well as with sphere-forming ability but its overexpression was reduced in a mutant p53 context. PARP expression increased with advanced dedifferentiation but not in the p53 mutants. *** DNA-PKcs. Somatic mutations in DNA-PKcs have been identified in patients with breast and pancreatic cancer. Ablation of mouse Thr2605 leads to multiple stem cell defects and increased chromosomal alterations. ^ Human CtIP. CtIP is a multifunctional protein that interacts with multiple partners, including the oncogenic corepressor CtBP, tumor suppressor RB, promoter of cell proliferation Pin1, and oncogene and DNA repair protein BRCA1. It is involved in the activation of the G2/M cell cycle checkpoint and in collaboration with the MRN complex 5′-3′ resection of broken DNA ends. CtIP deficiency impairs DSB repair. However, in contrast to BRCA1, CtIP is amplified in a substantial proportion of pancreatic cancer, high-grade serous ovarian cancer, esophageal carcinoma, and gastric adenocarcinoma. Germline mutations have been identified in patients with early-onset breast cancer who are negative for BRCA1/2 mutations. However, some functions of CtIP could counteract tumorigenesis, and it was initially postulated to be a tumor suppressor based on the shortened lifespan and tumor predisposition of ctip^+/−^ heterozygous mice. In short, CtIP plays very intricate roles in both DNA response and tumorigenesis but its involvement in telomere homeostasis has been demonstrated.

**Table 2:** Components of the DDR which are responsible for DNA repair syndromes that manifest early in life as clinical entities and are concurrently associated with cancer predisposition and telomere homeostasis.

	Telomere Dysfunction	Cancer Predisposition
(See comment) ERCC6like2 (ERCC6L2)	Yes [57]	Yes [46]
CMMRD-Xeroderma pigmentosum	Yes [58]	Yes [33,46]
Bloom syndrome	Yes [59]	Yes [46,59]
Werner syndrome	Yes [59]	Yes [46,59]
Rothmund–Thomson syndrome	Yes [60]	Yes [46]

Note regarding Table 2. Ref. [49] described three new cases of trilineage bone marrow failure, microcephaly, and learning difficulties consistent with ERCC6L2 deficiency. Exome sequencing identified homozygous variants of ERCC6L2 in two cases. Telomere shortening was present in two.

**Table 3:** Common and differential features of ataxia-telangiectasia mutated, ataxia-telangiectasis-like disease, and Nijmegen breakage syndrome.

ATM-Mutated	Mer11 (AT-LD)	NBN (Nijmegen Breakage Syndrome) (NBS)
Deficient initial DNA repair.	Deficient initial DNA repair.	Deficient initial DNA repair.
Hypersensitivity to IR-induced chromosome breakage, increased translocations (chromosomal instability).	Hypersensitivity to IR-induced chromosome breakage, increased translocations (chromosomal instability).	Hypersensitivity to IR-induced chromosome breakage, increased translocations (chromosomal instability).
IR-induced thymocyte apoptosis relative to wild-type: 58% versus 90%. Severe intra-S and G2/M checkpoint defects. Telomere shortening. Cancer predisposition.	IR-induced thymocyte apoptosis relative to wild-type: Sightly reduced (81% versus 90%. Intra-S and G2/M checkpoints less severe than in ATM deficiency and comparable to Nsb1 null. No telomere shortening. No cancer predisposition (or mild).	IR-induced thymocyte apoptosis: Extreme variations in apoptotic capacity (in neural tissue similar to wild-type). Intra-S and G2/M checkpoints less severe than in ATM deficiency and comparable to Mre11 hypomorphic. Telomere shortening. Cancer predisposition.

## Data Availability

All material used for this study is within the list of references.
